# Supplementary material for: Sleep fMRI with simultaneous electrophysiology at 9.4 T in male mice
Source: Nat Commun. 2023 Mar 24;14:1651. doi: 10.1038/s41467-023-37352-9 (PMC10039056; doi:10.1038/s41467-023-37352-9)
Supplement: Supplementary file 1 — Supplementary Information [file 41467_2023_37352_MOESM1_ESM.pdf]

Supplementary information

Title: Sleep fMRI with simultaneous electrophysiology at 9.4T in male mice

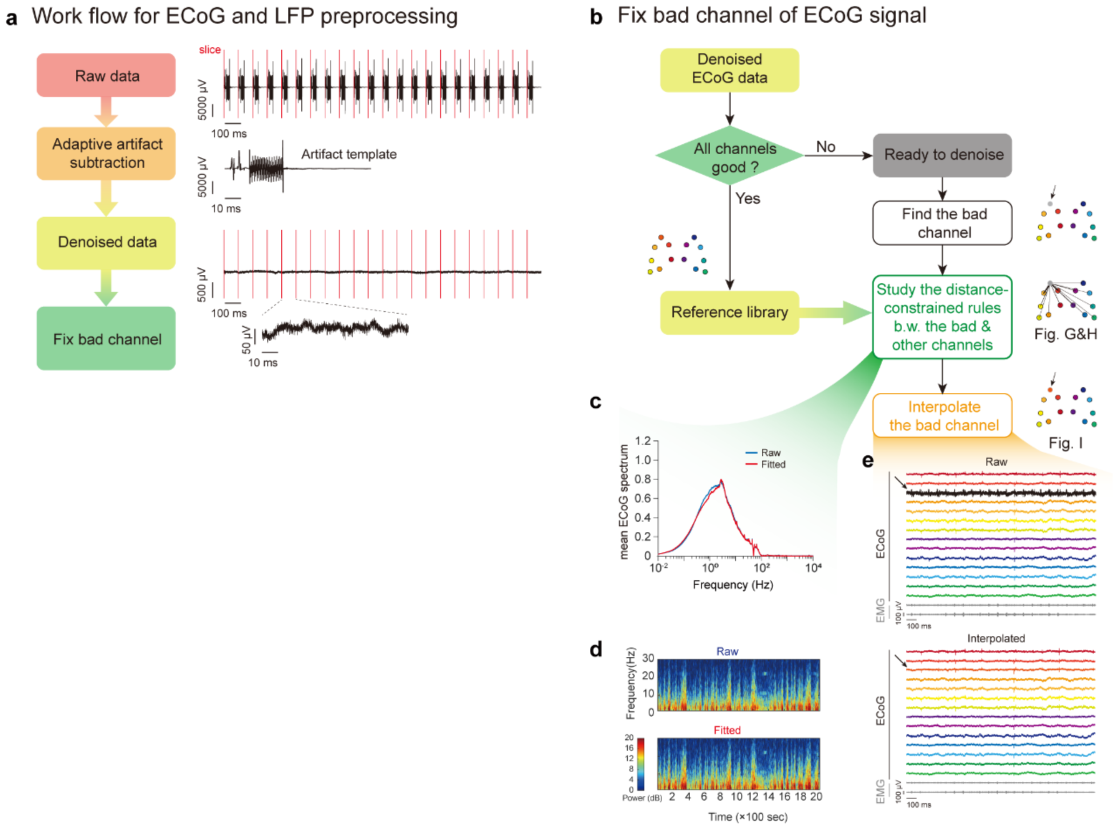

Supplementary Figure 1 | Related to Main Figure 2

Quality control of electrophysiological signals.

(a) Work flow for the quality control of electrocorticogram (ECoG) and local field potential (LFP) signals. The off-line correction of MRI gradient artifacts in the electrophysiological signals was conducted using the fMRI Artifact Slice Template Removal algorithm (FASTR). Details were described in the Method part.

(b) Pipeline for interpolating the signal of abnormal ECoG channel(s). Details were described in the Method part.

(c) Similar power spectrums between raw noisy and fitted ECoG signals.

(d) Similar time-frequency spectrums between raw noisy and fitted ECoG signals.

(e) Representative traces of raw and interpolated ECoG signals. Black arrow indicated the channel with abnormal noisy ECoG signals.

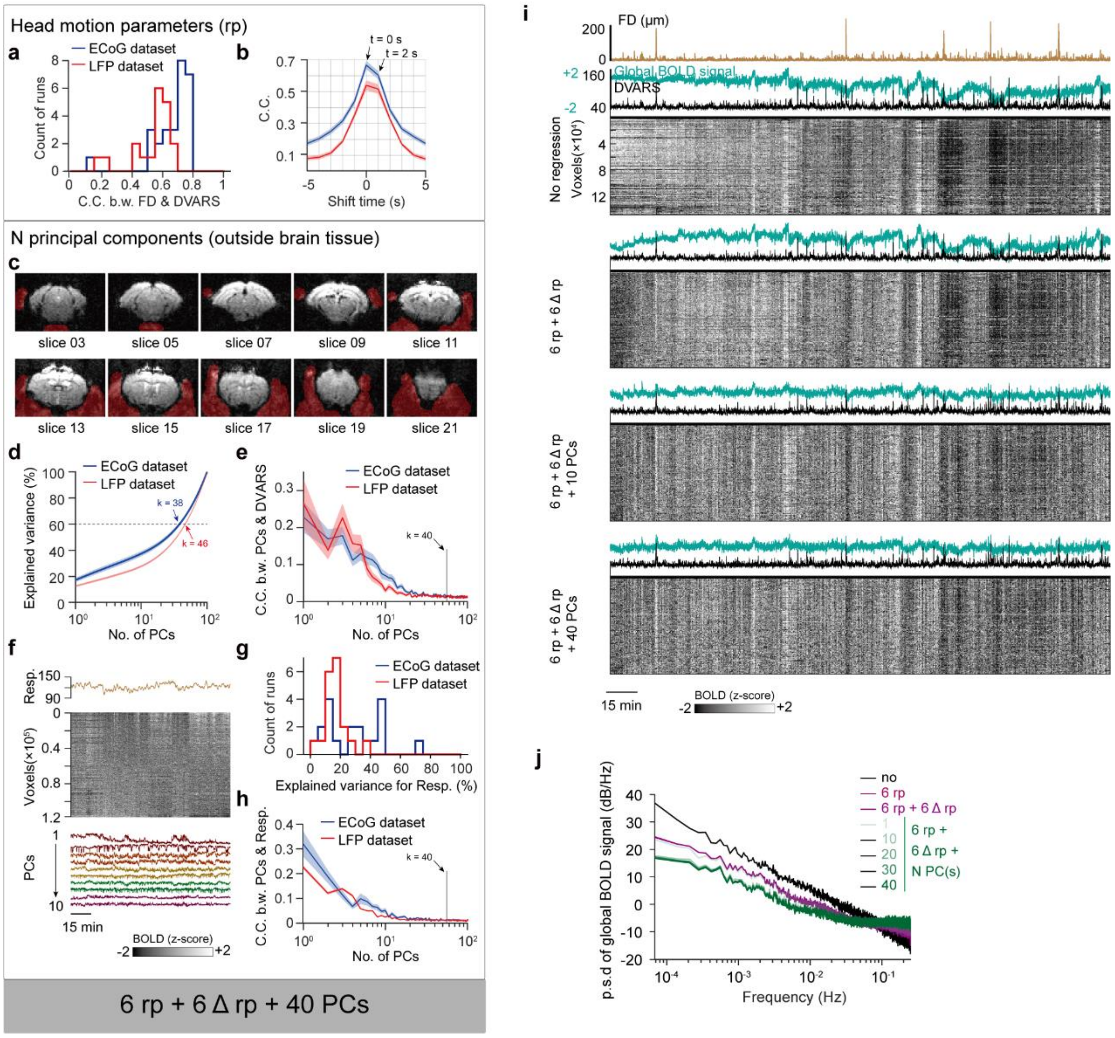

Supplementary Figure 2 | Related to Main Figure 2

Quality control of BOLD fMRI signals.

(a-h) The “6 rp + 6 Δrp + 40 PCs” highly covered the head motion, infra-slow drift of BOLD signals and other potential physiological noises.

(a) High Pearson’s correlation coefficients (C.C.) between frame-wise displacement (FD) and DVARS of raw BOLD signals.

(b) Time-shifted correlation between FD and DVARS. High correlation between FD and DVARS (shift time 2 s) indicated a rapid head motion influenced BOLD signals of the current and the next EPI volumes. Colored lines (or shadows), mean (+/- SEM.) correlation.

(c) Representative mask (red shadows) used for extracting the principal components (PCs) outside mouse brain tissue to model non-neural signal variations.

(d) Approximate 40 PCs captured 60% of the signal variance in the tissues outside the brain.

(e) High Pearson’s correlations between DVARS and PCs from non-neural signals. Colored lines (or shadows), mean (+/- SEM.) correlation.

(f) Representative example of mouse respiration rate (upper panel), voxel-wise BOLD time series (middle panel) and top 10 non-neuronal PCs (lower panel).

(g) Mouse respiration rate could be highly captured by the top 40 non-neural PCs.

(h) High Pearson’s correlations between respiration rate and top 40 non-neural PCs. Colored lines

(or shadows), mean (+/- SEM.) correlation.

(i-j) Regression of "6 rp + 6 Δrp + 40 PCs" notably reduced the impact of nuisance signals on whole brain BOLD signals.

(i) Visualization of effects of nuisance signal regression. Representative data with head motion and resulting signal variations. The time courses of whole brain voxels were shown as intensity plots.

(j) Minimal infra-slow drift of BOLD global signal after the "6 rp + 6 Δrp + 40 PCs" regression. p.s.d., power spectral density.

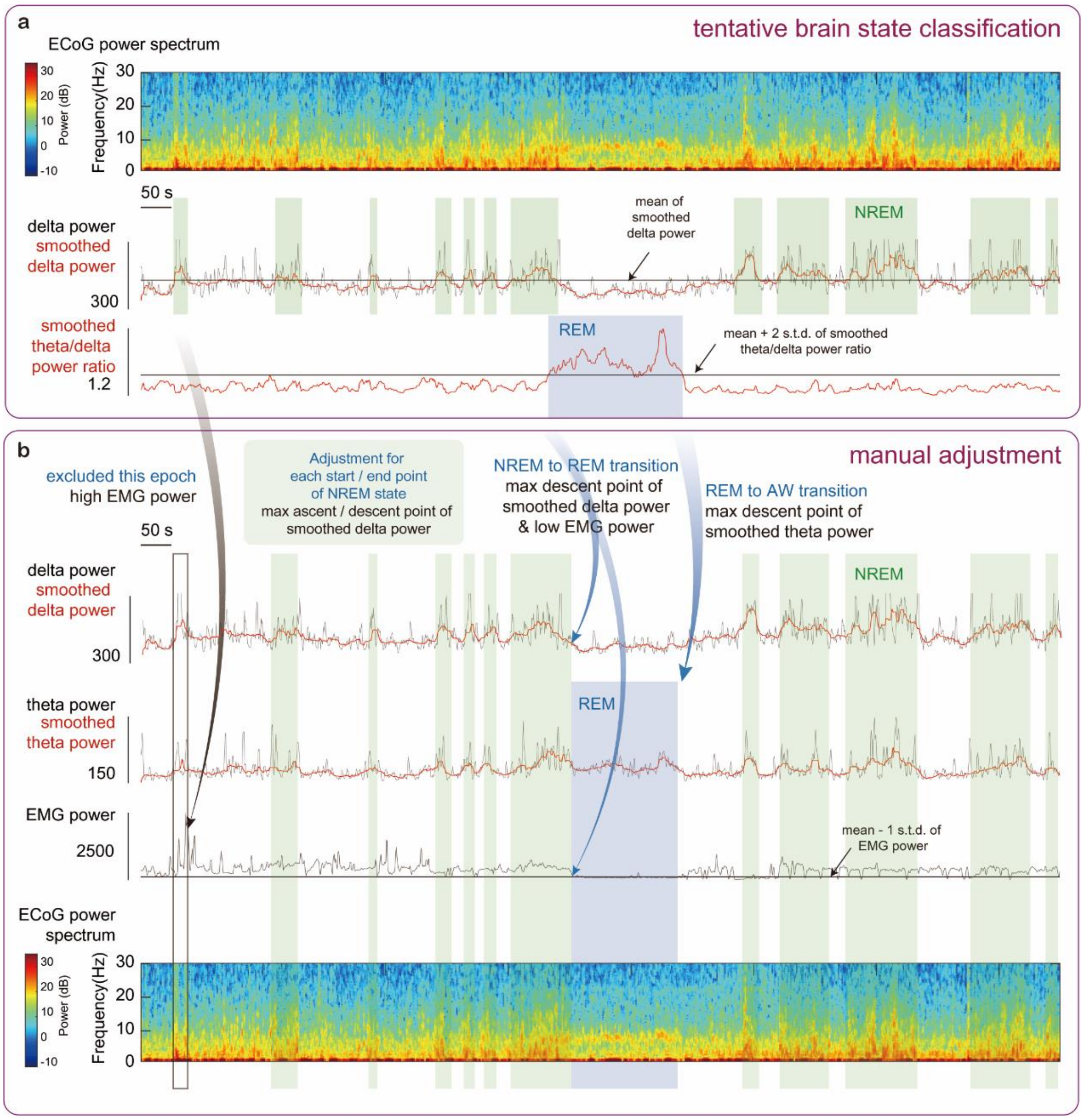

Supplementary Figure 3 | Related to Main Figure 2

Example of brain states classification procedure (session 1: channel 10, 11500-13000s).

(a) Tentative brain state classification. A time point was classified as NREM sleep if the delta power was higher than its mean; a time point was assigned as REM sleep if the theta/delta power ratio was two standard deviations higher than its mean and all remaining time points were classified as AW state.

(b) Manual adjustment. For NREM state, according to the ECoG channel power spectrum, we adjusted the start or end point to the point with the greatest ascent or descent speed of smoothed delta power. For REM state, the start point was adjusted to the end point of the previous NREM, and the end point was adjusted to the point with the greatest descent speed of smoothed theta power. If the greatest ascent or descent point of EMG power was different from that of ECoG/iEEG signals, the midpoint between the two was defined as the transition point. High EMG power NREM epoch was manually excluded. Source data are provided as a Source Data file.

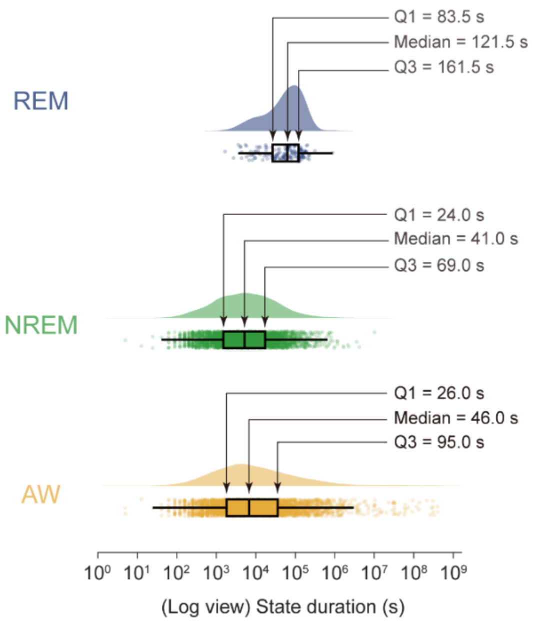

Supplementary Figure 4 | Related to Main Figure 2

Distributions of AW, NREM and REM state duration.

Distributions of awake (AW), non-rapid eye movement (NREM) and rapid eye movement (REM) state duration (AW: 3872 epochs, NREM: 3851 epochs, REM: 168 epochs, n=46 sessions). Each dot represented an epoch of corresponding brain state. On each box plot, the central mark indicated the mean, and the bottom and top edges of the box indicated the 25th and 75th percentiles, respectively. The whiskers extend to the most extreme data points not considered outliers. Colored shade represented the probability of state durations. Q1, lower quartile; Q3, upper quartile. Source data are provided as a Source Data file.

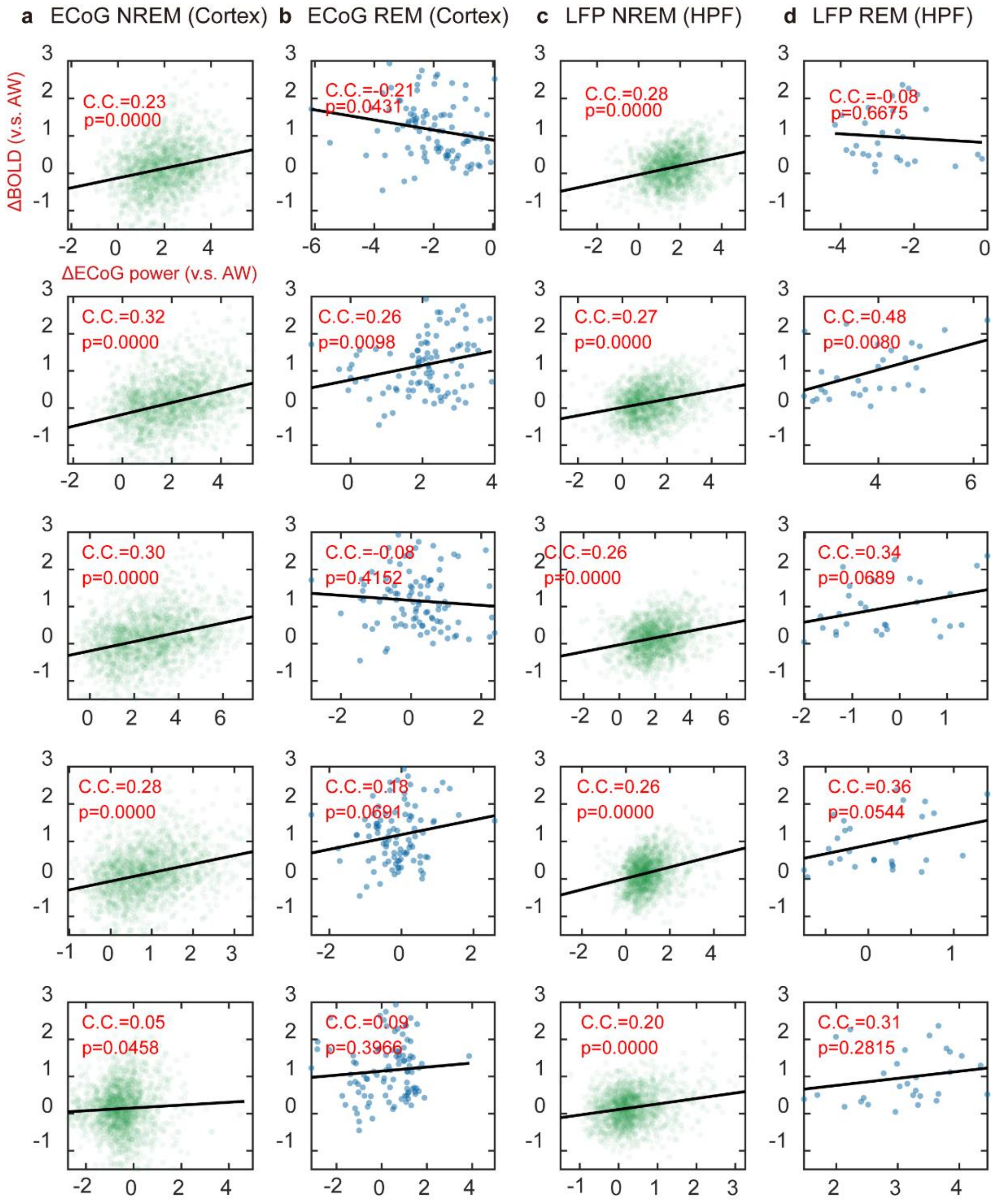

Supplementary Figure 5 | Related to Main Figure 3

Electrophysiological correlates of sleep evoked BOLD responses.

(a-b) Pearson's correlation coefficients (C.C.) between cortical relative changes of ECoG band-limited power and BOLD responses in NREM (a) and REM (b), compared to AW state. Statistical significance was calculated by two-tailed t-test. Each dot, an epoch of corresponding state. Black line, the best linear fit. delta (δ), 1-4 Hz; theta (θ), 5-10 Hz; alpha (α), 11-20 Hz; beta (β), 21-40 Hz; gamma (γ), 41-100 Hz.

(c-d) As in (a-b) but for LFP signals in hippocampus (HPF). For ECoG dataset, the sample size of NREM is 2372 and REM is 138. For LFP dataset, the sample size of NREM is 1479 and REM is 30. Statistical significance was calculated by two-tailed t-test. Source data are provided as a Source Data file.

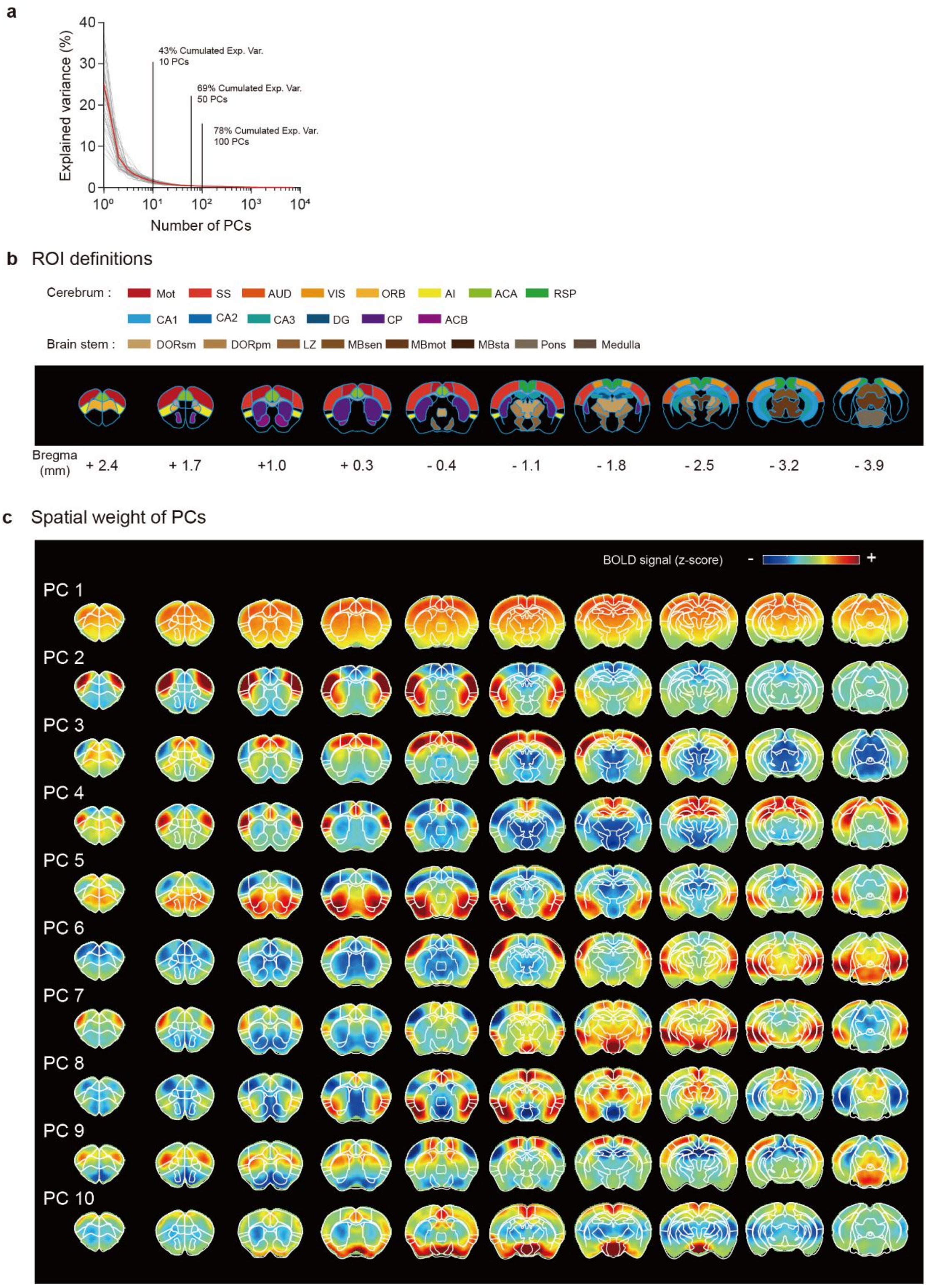

Supplementary Figure 6 | Related to Main Figure 4

Group principal component analysis of BOLD fMRI signals.

(a) Explained variance accounted by principal components (PCs). The first 4 PCs accounted for at least 3 % variance. Gray line represented the explained variance of each individual EPI scan. Black line represents the averaged explained variance using the group principal component analysis (PCA) strategy.

(b) Region-of-interest (ROI) definitions derived from CCFv3 Allen mouse brain atlas. Abbreviations were list in Supplementary Data 1.

(c) Spatial maps for the first 10 PCs (colored according to spatial weight; no threshold was applied). More spatial profiles of PCs (top 100) were shown in Supplementary Data 2.

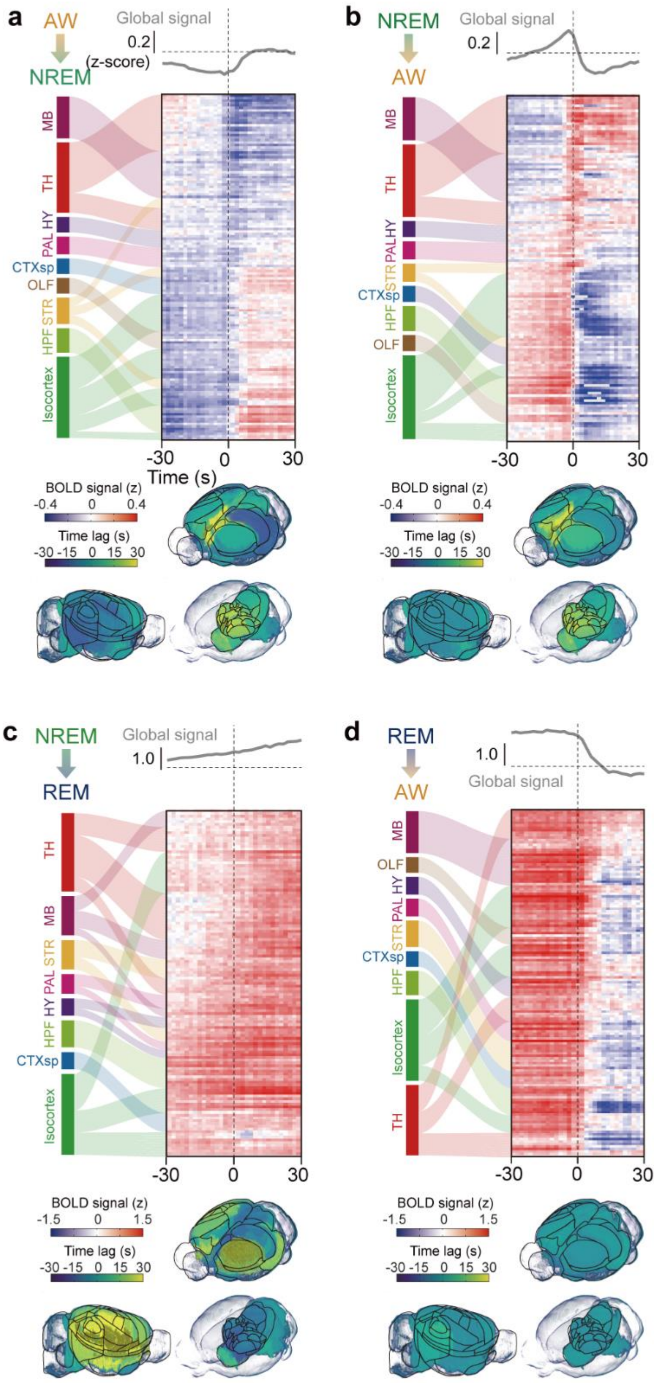

Supplementary Figure 7 | Related to Main Figure 4

Sequential BOLD signal fluctuations traversing the mouse brain during brain state transitions, Including "AW to NREM" (a), "NREM to AW" (b), "NREM to REM" (c) and "REM to AW" (d)**.**

Upper panel, dynamics of global BOLD signal. ("AW to NREM", n=1803 epochs; "NREM to AW", n= 1720 epochs; "NREM to REM", n= 140 epochs; "REM to AW", n= 128 epochs).

Middle panel, time-region graphs of BOLD signal synchronized to electrophysiological signal defined state transitions (time 0). Quantitative results were shown in Supplementary Data 3. Detailed spatiotemporal maps across state transitions were shown in following Supplementary Fig. 8-11 in coronal slice view.

Lower panel, time delay relative to the global signal displayed on a 3D surface. Detailed time lag

profiles were shown in following Supplementary Fig. 12 in coronal slice view. Source data are provided in Supplementary Data 3.

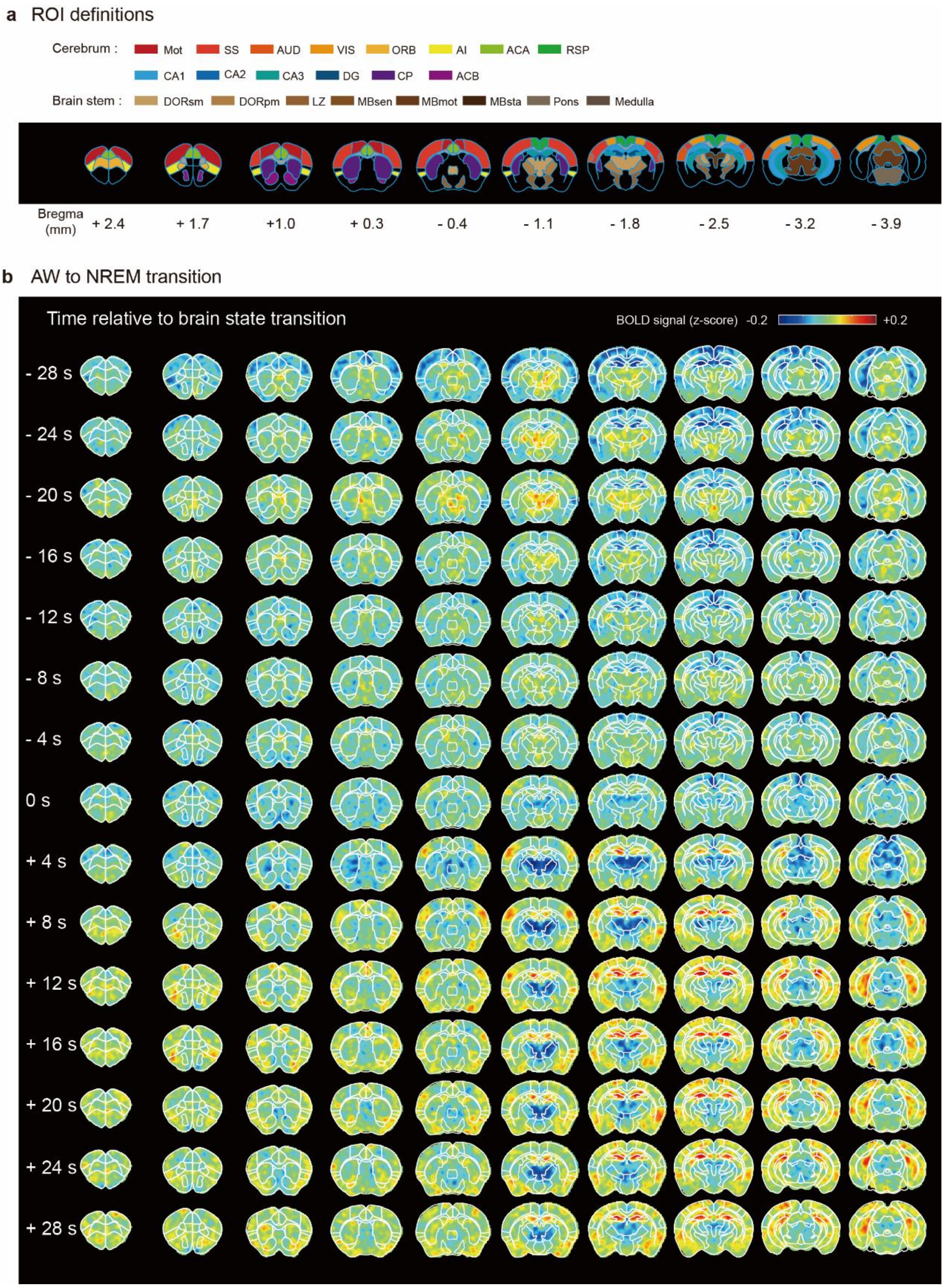

Supplementary Figure 8 | Related to Main Figure 4

Spatiotemporal dynamics of BOLD signals along with "AW to NREM" state transition.

(a) ROI definitions derived from CCFv3 Allen mouse brain atlas. Abbreviations were list in Supplementary Data 1.

(b) Spatiotemporal BOLD signal variations along with "AW to NREM" state transition (n=1803 epochs). Quantitative results were shown in Supplementary Data 3.

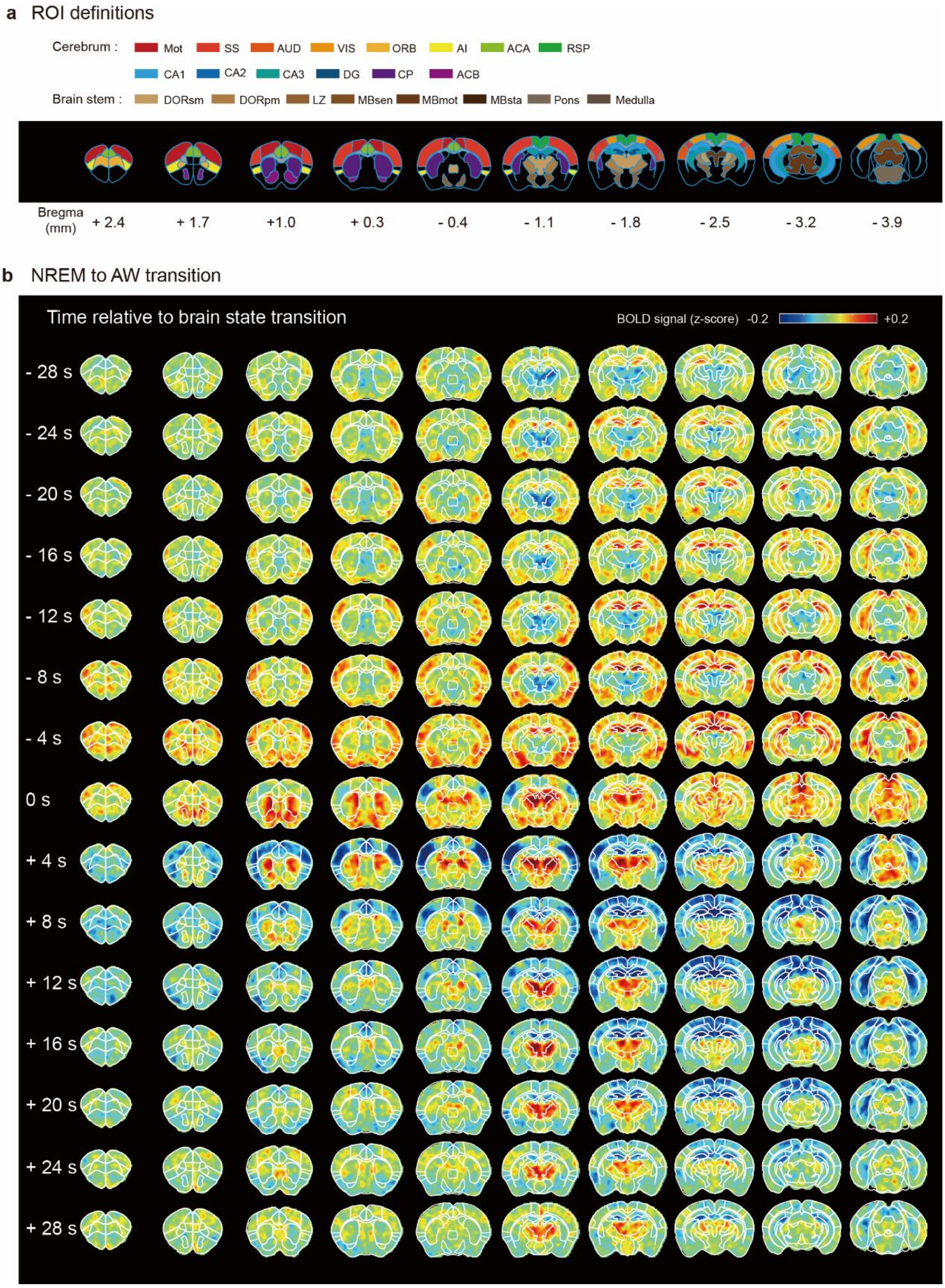

Supplementary Figure 9 | Related to Main Figure 4

Spatiotemporal dynamics of BOLD signals along with "NREM to AW" state transition.

(a) ROI definitions derived from CCFv3 Allen mouse brain atlas. Abbreviations were list in Supplementary Data 1.

(b) Spatiotemporal BOLD signal variations along with "NREM to AW" state transition (n=1720 epochs). Quantitative results were shown in Supplementary Data 3.

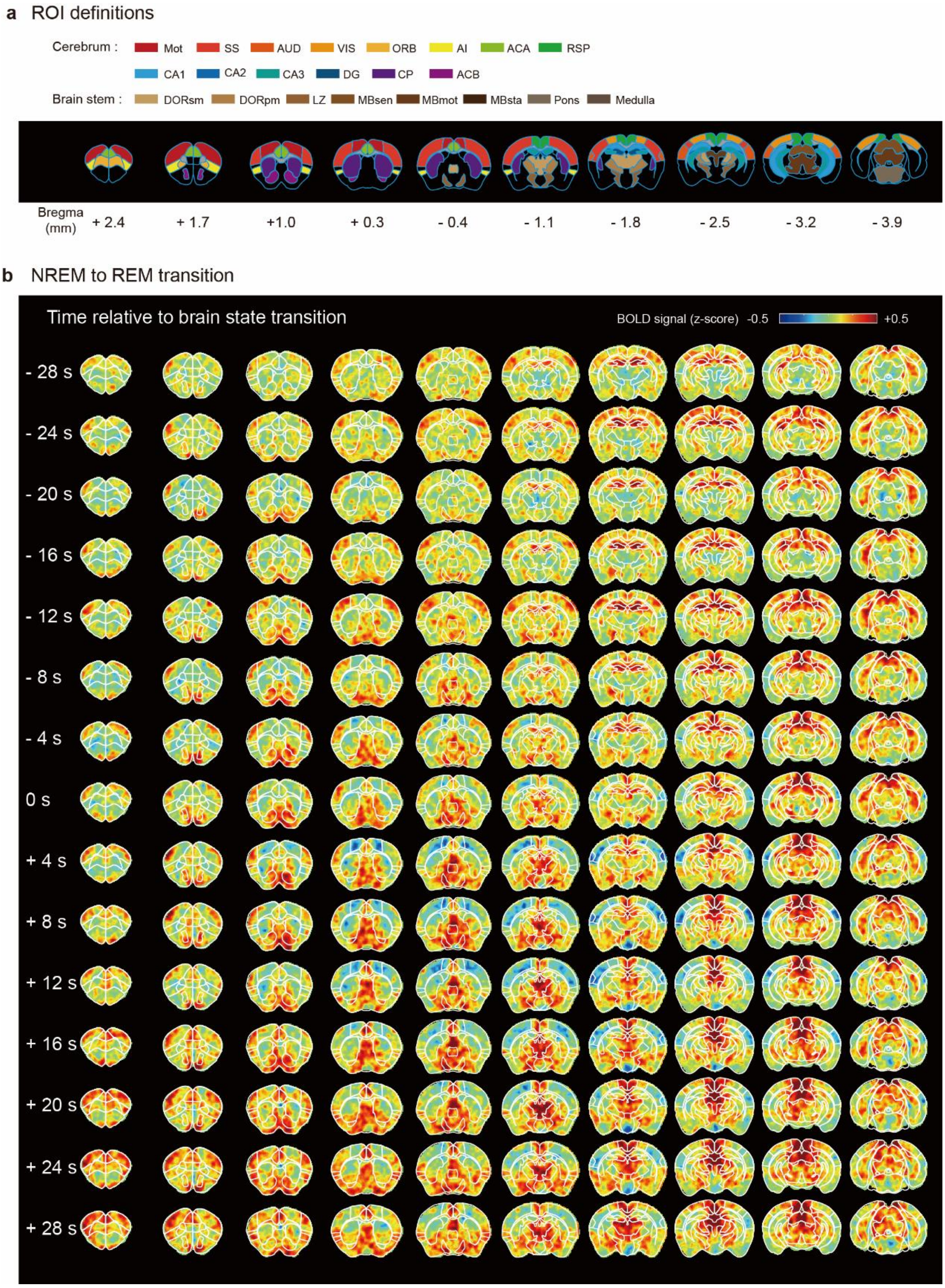

Supplementary Figure 10 | Related to Main Figure 4

Spatiotemporal dynamics of BOLD signals along with "NREM to REM" state transition.

(a) ROI definitions derived from CCFv3 Allen mouse brain atlas. Abbreviations were list in Supplementary Data 1.

(b) Spatiotemporal BOLD signal variations along with "NREM to REM" state transition (n=140 epochs). Quantitative results were shown in Supplementary Data 3.

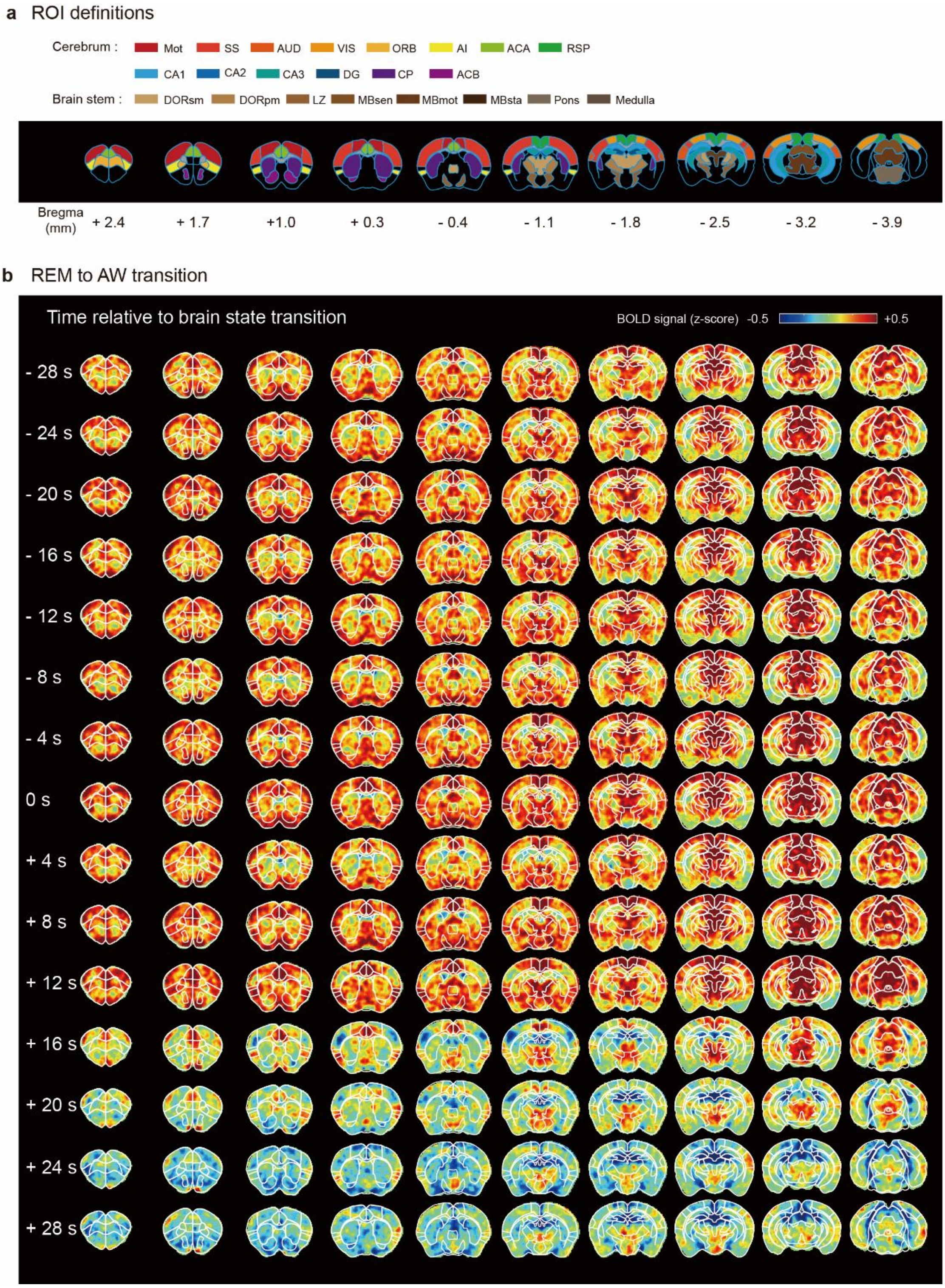

Supplementary Figure 11 | Related to Main Figure 4

Spatiotemporal dynamics of BOLD signals along with "REM to AW" state transition.

(a) ROI definitions derived from CCFv3 Allen mouse brain atlas. Abbreviations were list in Supplementary Data 1.

(b) Spatiotemporal BOLD signal variations along with "REM to AW" state transition (n=128 epochs). Quantitative results were shown in Supplementary Data 3.

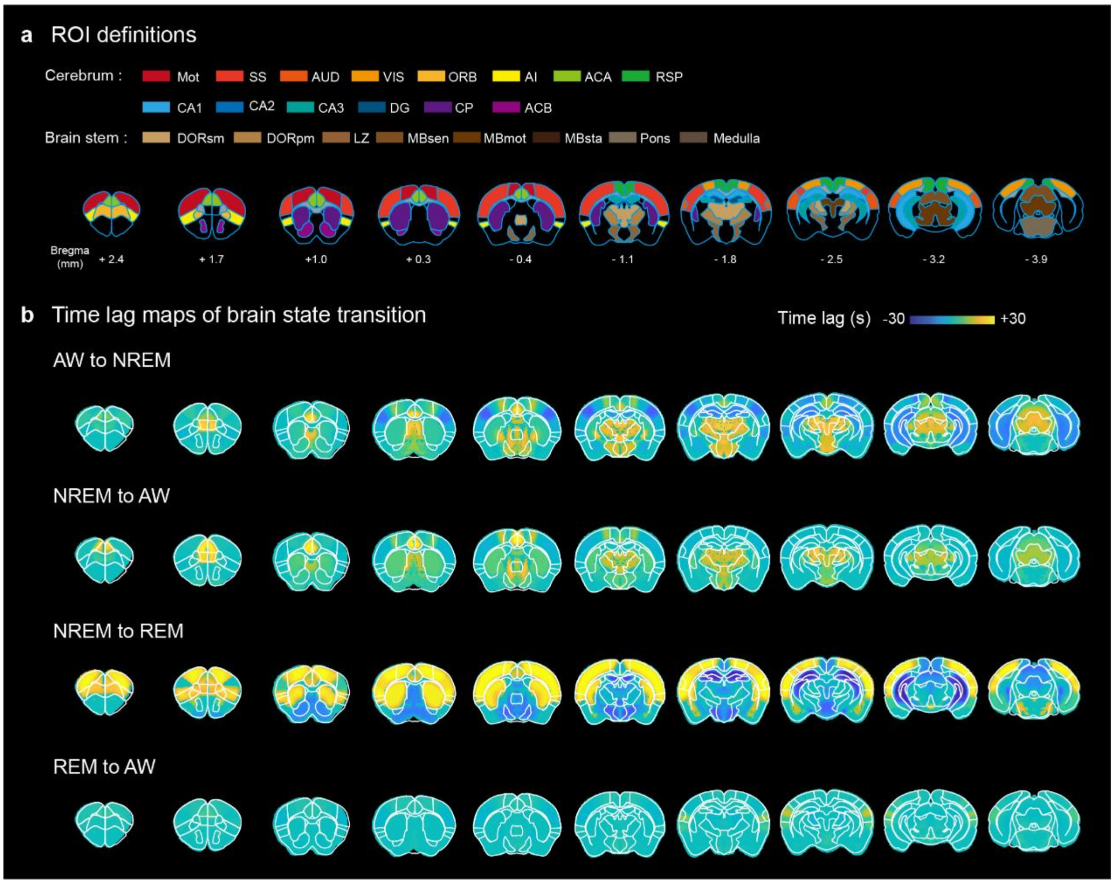

Supplementary Figure 12 | Related to Main Figure 4

Time Lag profiles of whole brain BOLD signals along with brain state transitions.

(a) ROI definitions derived from CCFv3 Allen mouse brain atlas. Abbreviations were list in Supplementary Data 1.

(b) Time Lag profiles of whole brain BOLD signals along with brain state transitions. No threshold was applied.

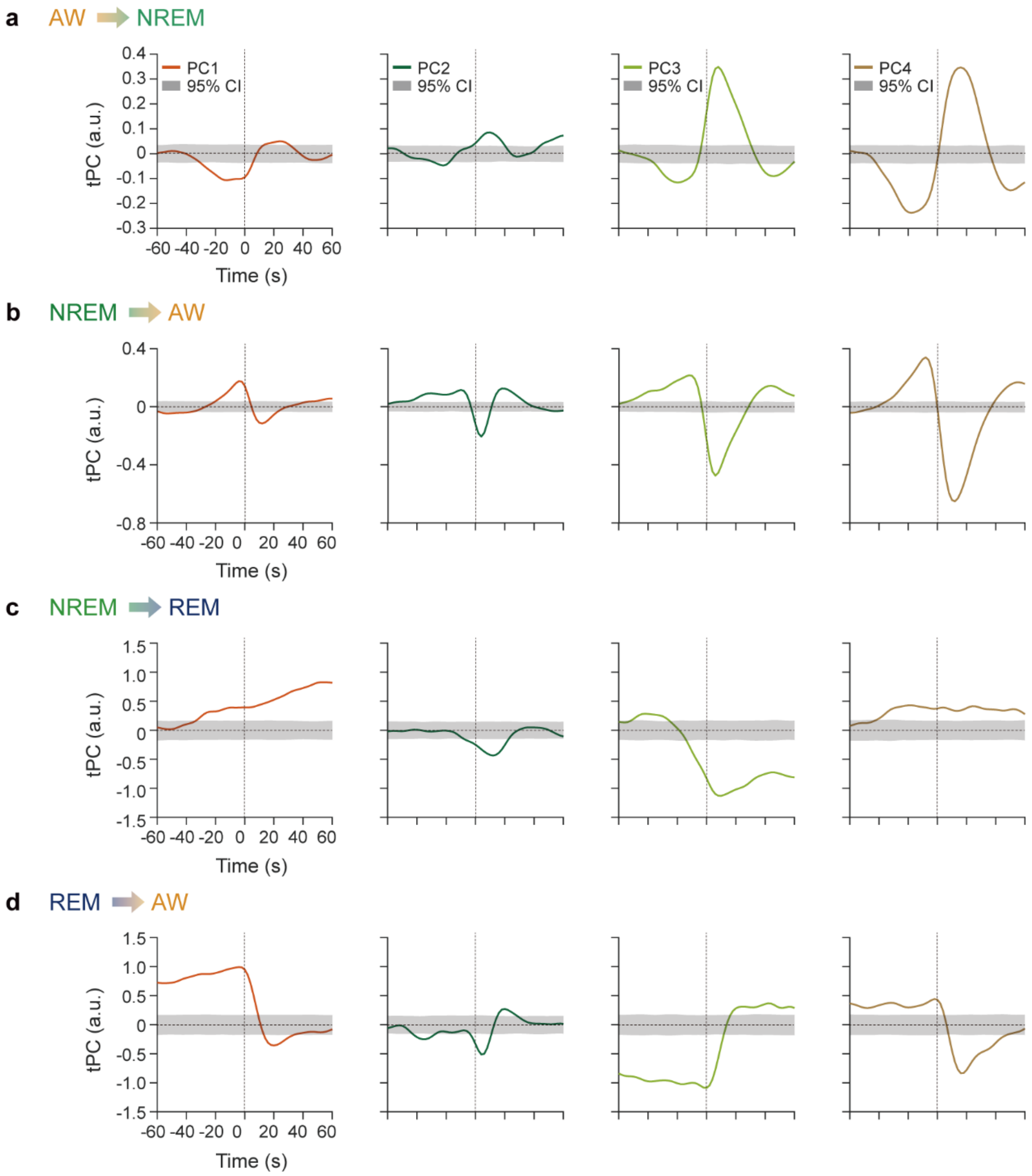

Supplementary Figure 13 | Related to Main Figure 4

Quantitative evaluations of temporal weights dynamic across brain state transitions.

(a-d) The temporal weights during "AW to NREM" (a), "NREM to AW" (b), "NREM to REM" (c) and "REM to AW" (d) transitions. Color line, mean temporal weights of each PC (tPCs); gray shadow, the 95% confidence interval (CI) of null control. "AW to NREM", n=1803 epochs; "NREM to AW", n= 1720 epochs; "NREM to REM", n= 140 epochs; "REM to AW", n= 128 epochs. a.u. arbitrary unit. Source data are provided as a Source Data file.

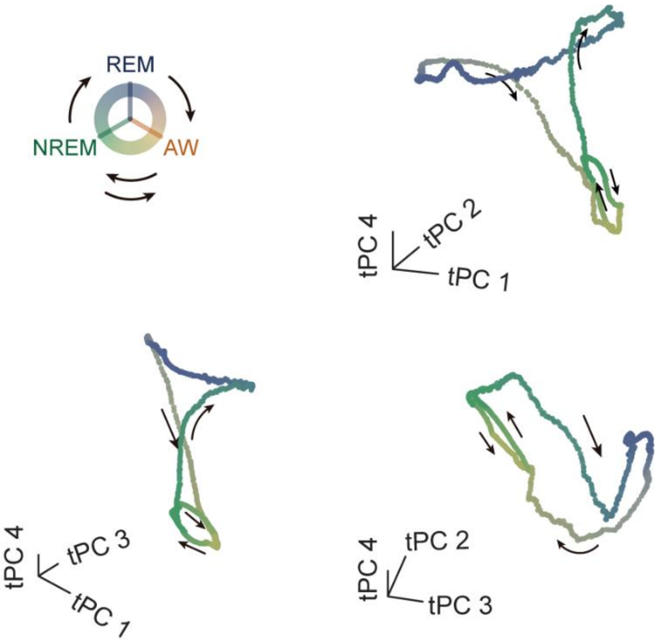

Supplementary Figure 14 | Related to Main Figure 4

Low dimensional manifold of BOLD signals traversed by the brain state across three PCs. Arrows, the directions of flow along the manifold.

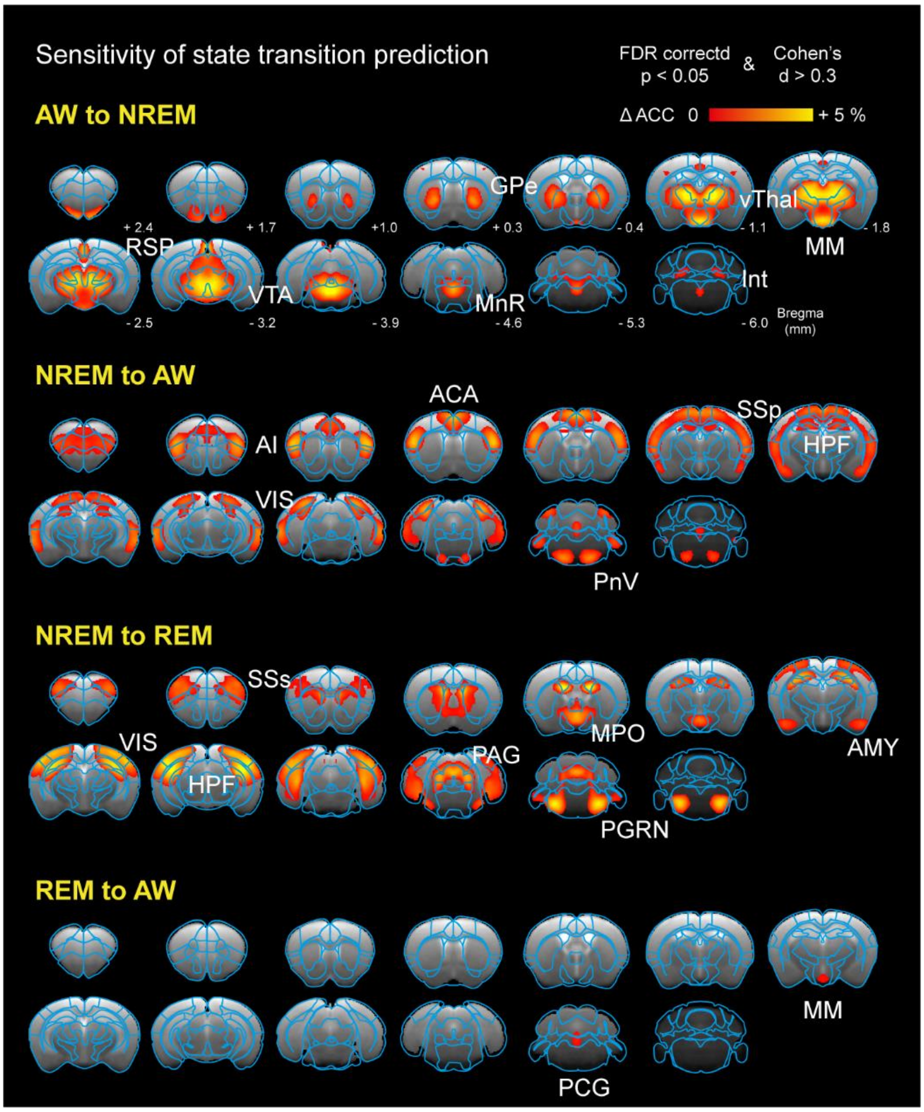

Supplementary Figure 15 | Related to Main Figure 5

Significantly sensitive regions of LSTM RNN models on brain state predictions

Sensitivity regions were shown in coronal view (one sample t-test, right tail). GPe, globus pallidus (external segment); vThal, ventral thalamus; MM, medial mammillary nucleus; RSP, retrosplenial area; VTA, ventral tegmental area; MnR, median raphe nucleus; Int, interposed cerebellar nucleus; AI, agranular insular area; ACA, anterior cingulate area; SSp, primary somatosensory area; HPF, hippocampal formation; VIS, visual areas; PnV, pontine reticular nucleus (ventral part); SSs, supplemental somatosensory areas; MPO, medial preoptic area; AMY, amygdala areas; PAG, periaqueductal gray; PGRN, paragigantocellular reticular nucleus; PCG, pontine central gray.

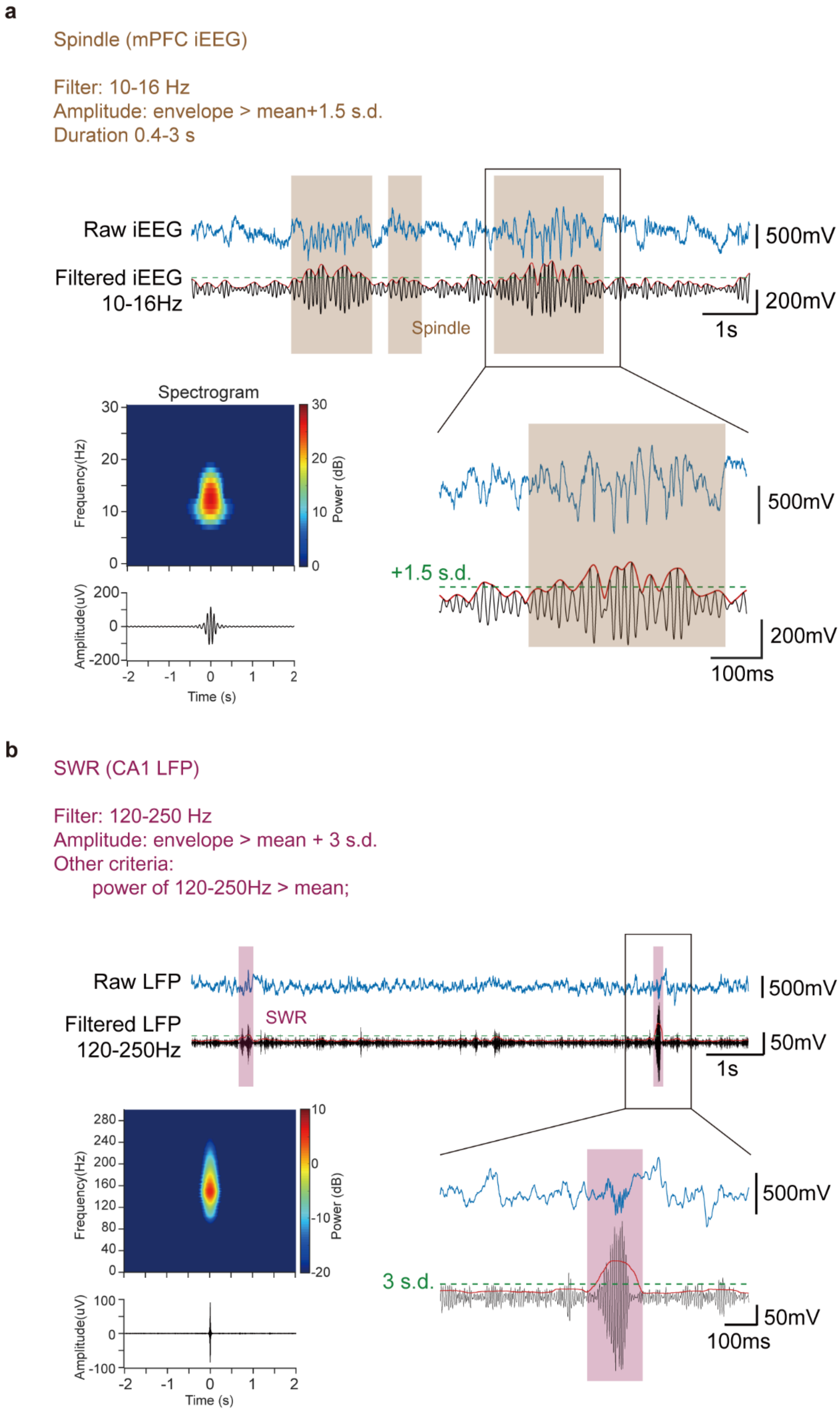

Supplementary Figure 16 | Related to Main Figure 6

Event identifications of spindle and sharp wave ripple (SWR).

(a) Upper and right panels, to identify the spindle event, raw iEEG signals (blue curves) in mPFC

were bandpass filtered (black curves) with Butterworth filters (10~16 Hz). A spindle event was detected if the envelope (red curve) of the filtered iEEG signal larger than its mean + 1.5 s.d. (green dash line) in NREM state. Only spindles with 0.4~3 s durations were included. Brown shade represented the spindle event. Lower left panel, averaged power spectrogram and time series of spindle event.

(b) Similar to (a) but for the SWR event. Upper and right panels, to identify the SWR event, raw LFP signals (blue curves) in CA1 of hippocampus were bandpass filtered (black curves) with Butterworth filters (120~250 Hz). A SWR event was detected if (1) the envelope (red curve) of the filtered LFP signal larger than its mean + 3 s.d. (green dash line) and (2) the power of the filtered LFP signal larger than its temporal mean. Purple shade represented the spindle event. Lower left panel, averaged power spectrogram and time series of SWR event.

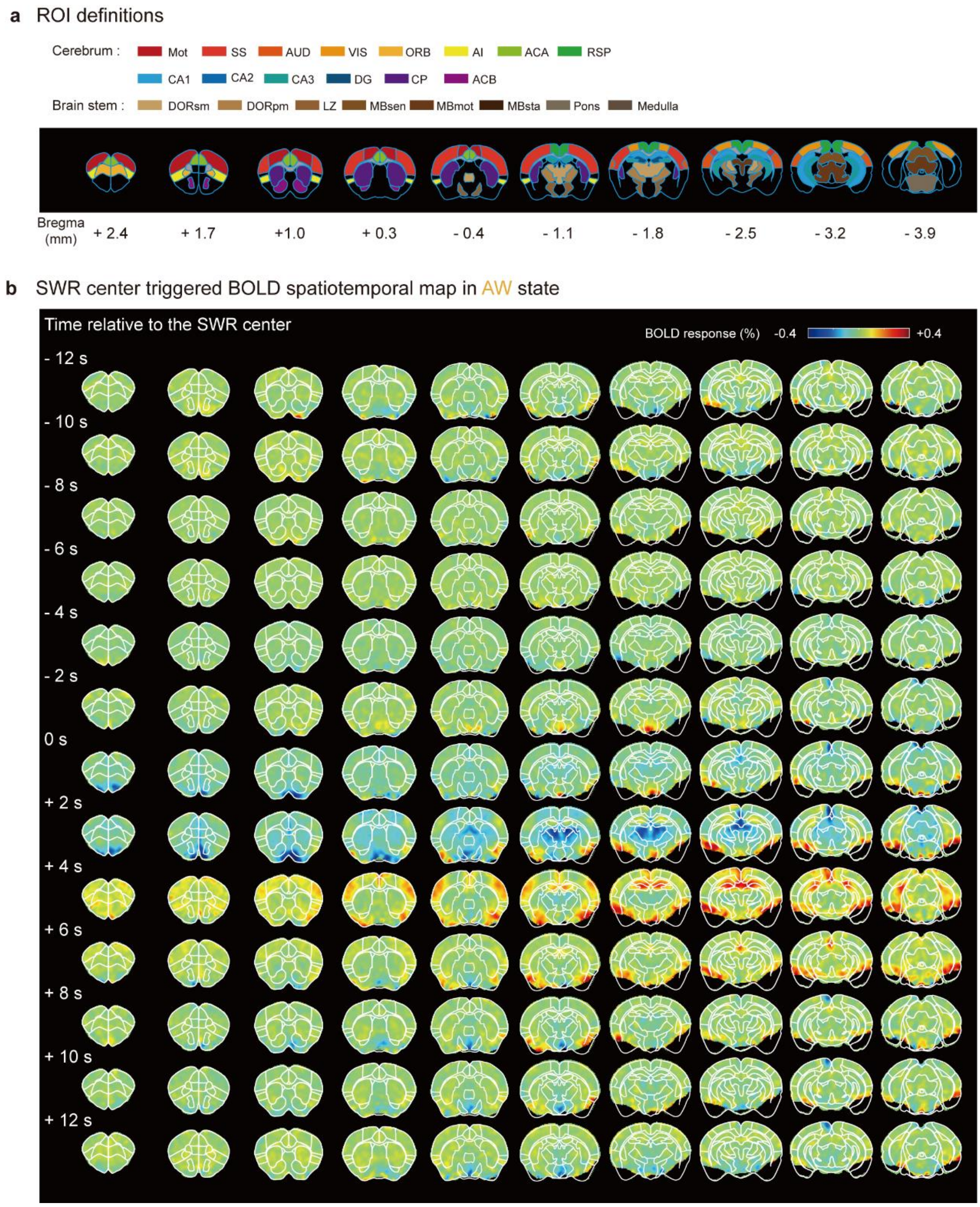

Supplementary Figure 17 | Related to Main Figure 7

SWR evoked BOLD signal dynamic in AW state.

(a) ROI definitions derived from CCFv3 Allen mouse brain atlas. Abbreviations were list in Supplementary Data 1.

(b) Spatiotemporal BOLD signals evoked by the SWR event in AW state (12732 epochs). The number of event epochs were counted under the sampling rate of 0.5 Hz (fMRI repetition time). More quantitative ROI-wise evaluation was shown in Supplementary Data 4.

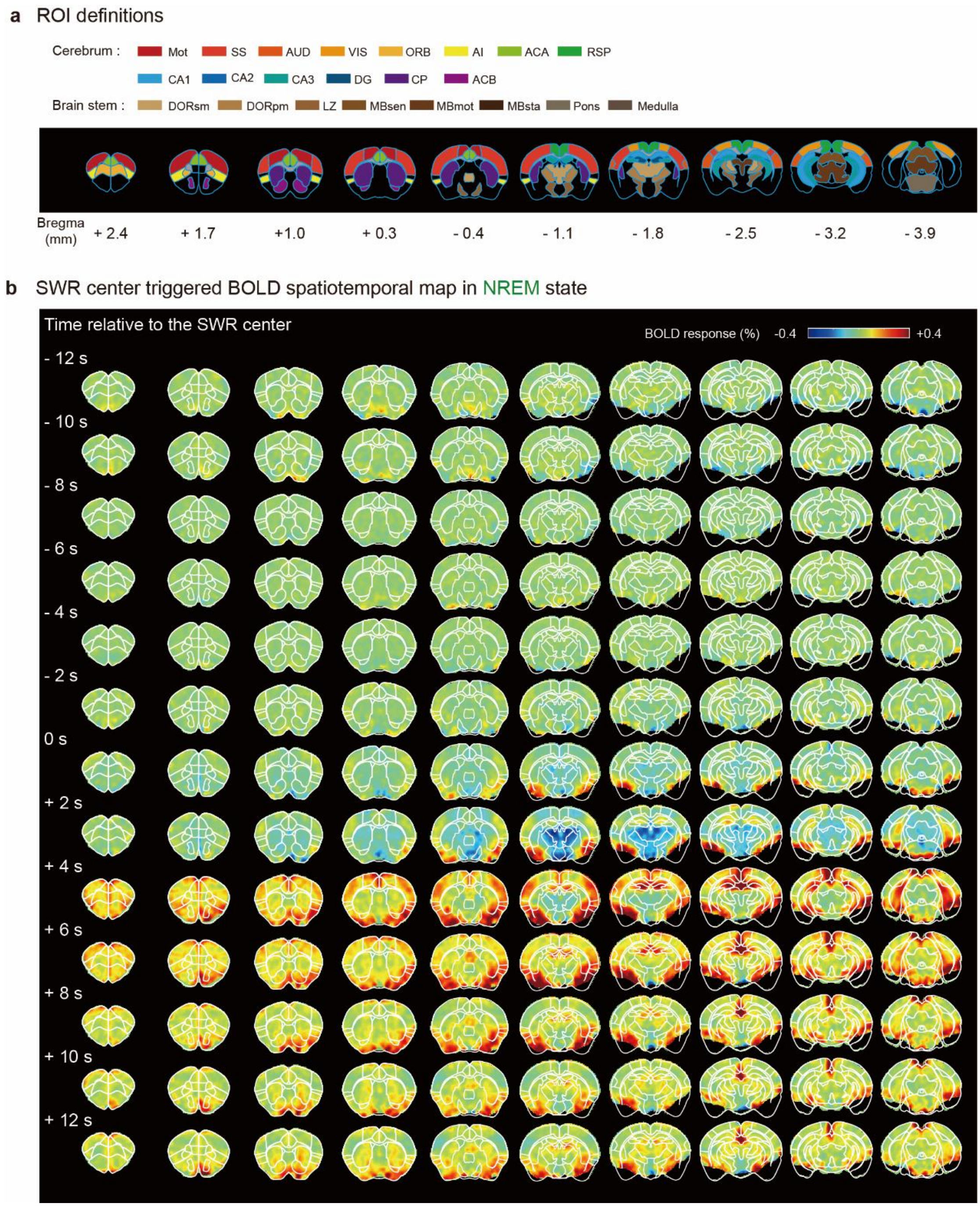

Supplementary Figure 18 | Related to Main Figure 7

SWR evoked BOLD signal dynamic in NREM state.

(a) ROI definitions derived from CCFv3 Allen mouse brain atlas. Abbreviations were list in Supplementary Data 1.

(b) Spatiotemporal BOLD signals evoked by the SWR event in NREM state (10953 epochs). The number of event epochs were counted under the sampling rate of 0.5 Hz (fMRI repetition time). More quantitative ROI-wise evaluation was shown in Supplementary Data 4.

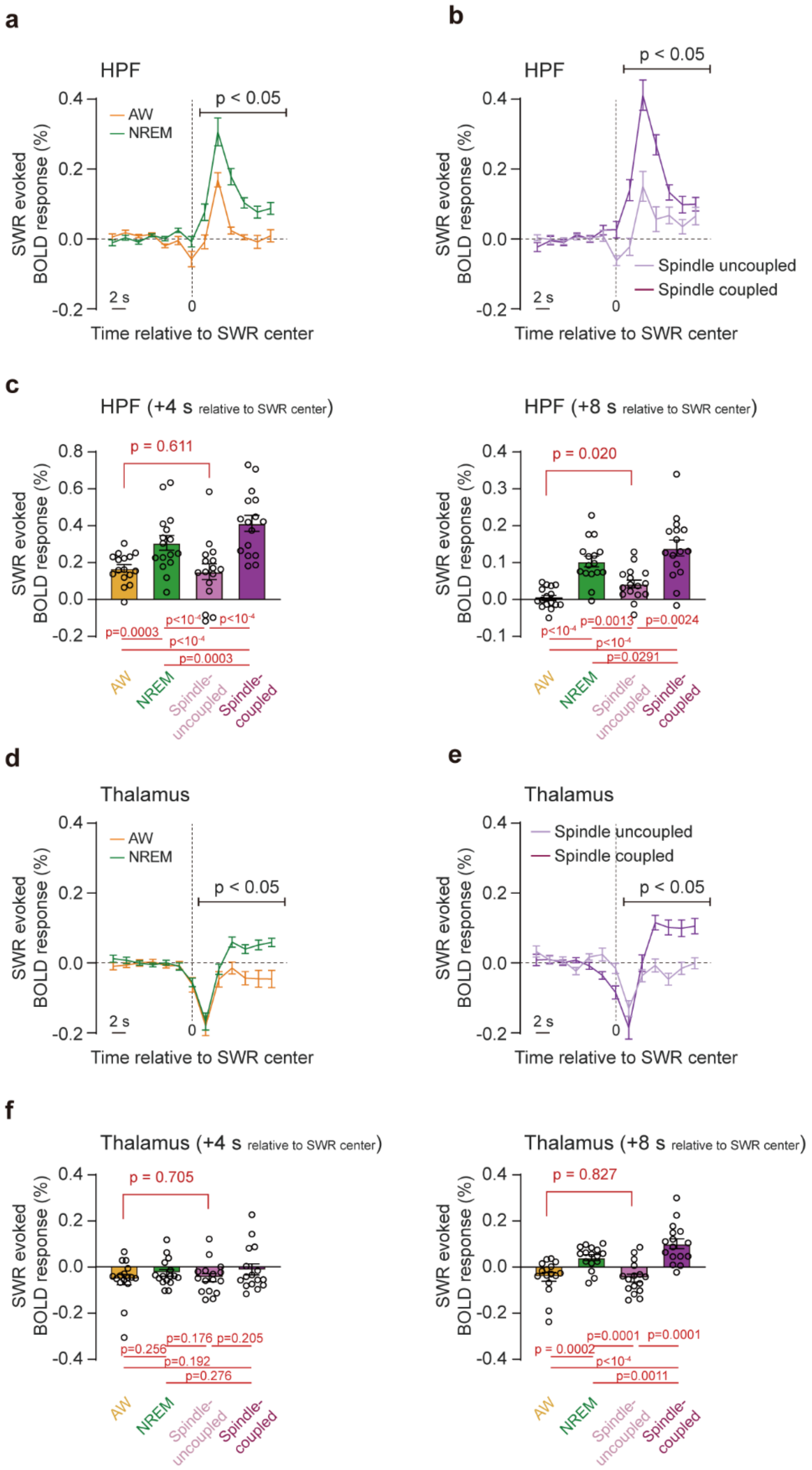

Supplementary Figure 19 | Related to Main Figure 7

SWR evoked BOLD signal dynamic in AW and NREM states.

(a) Significant difference of SWR evoked BOLD responses in HPF between AW and NREM state. HPF, hippocampus (n=16 sessions).

(b) Significant difference of event-evoked BOLD responses in HPF between spindle-uncoupled and coupled SWR in NREM state (n=16 sessions).

(c) AW SWR evoked BOLD signal in HPF were highly close to that of spindle-uncoupled SWR in

NREM state.

(**d-f**) As in (**a-c**) but for results in thalamus. n=16 sessions. Statistical significance was calculated by two-tailed t-test. Error bars, standard errors of the mean. Source data are provided as a Source Data file.

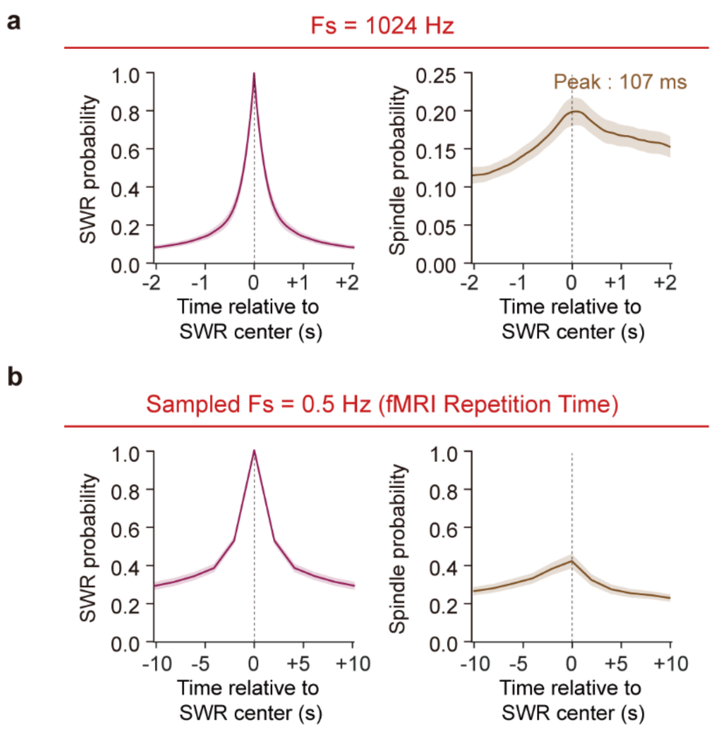

Supplementary Figure 20 | Related to Main Figure 7

Occurrence probability of SWR and spindle relative to SWR center.

(a) SWR triggered event occurrence probability of SWR (left panel) and spindle (right panel) at 1024 Hz sampling rate. Solid lines and shadows denoted the mean and std. of occurrence probability, respectively. Colored lines (or shadows), mean (+/- SEM.) event probability.

(b) Similar to (a) but sampled as 0.5 Hz (one fMRI repetition time). Colored lines (or shadows), mean (+/- SEM.) event probability.

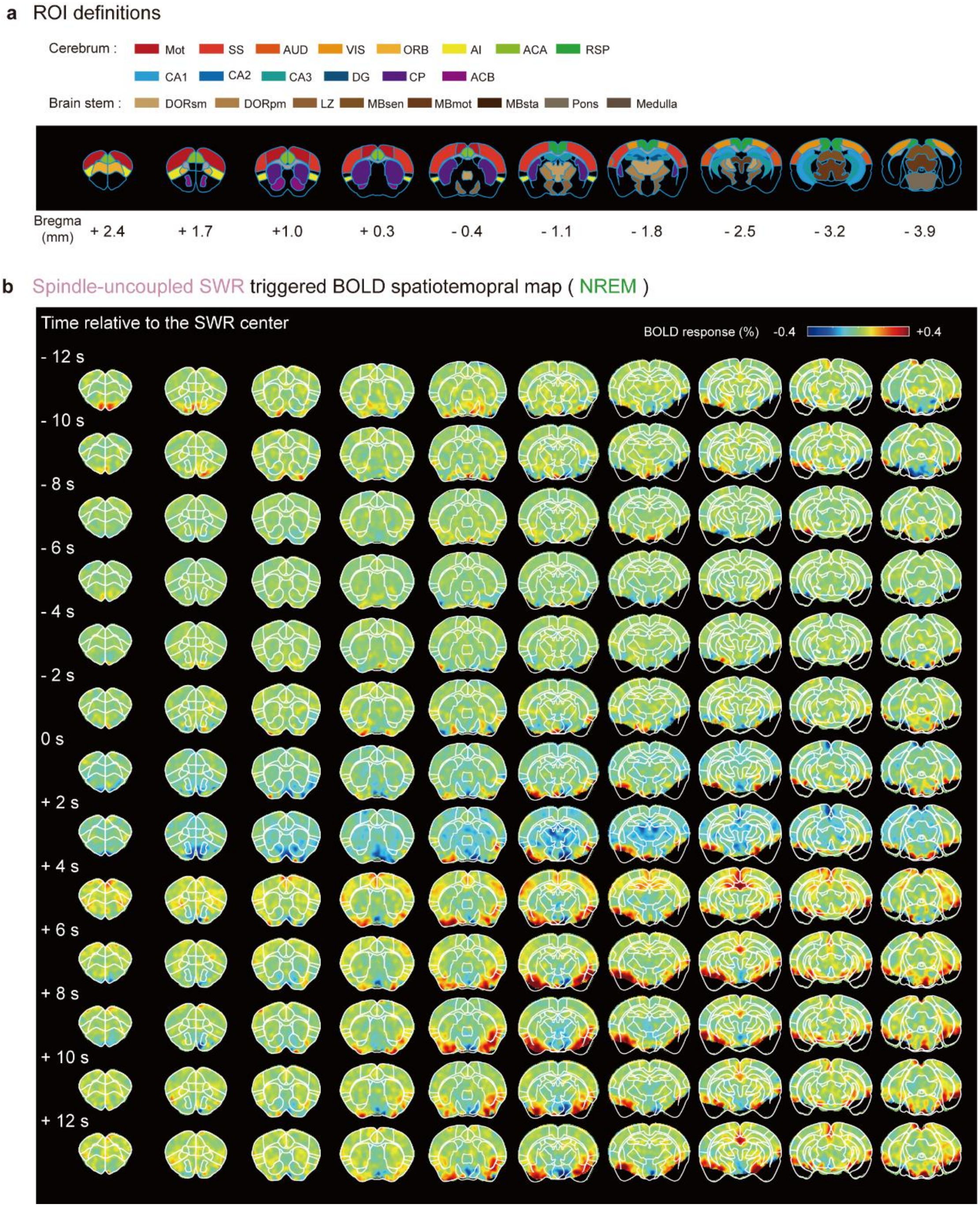

Supplementary Figure 21 | Related to Main Figure 7

Spindle-uncoupled SWR evoked BOLD signal dynamics in NREM state.

(a) ROI definitions derived from CCFv3 Allen mouse brain atlas. Abbreviations were list in Supplementary Data 1

(b) Spatiotemporal BOLD signals evoked by the spindle-uncoupled SWR event in NREM state (4682 epochs). The number of event epochs were counted under the sampling rate of 0.5 Hz (fMRI repetition time). More quantitative ROI-wise evaluation was shown in Supplementary Data 4.

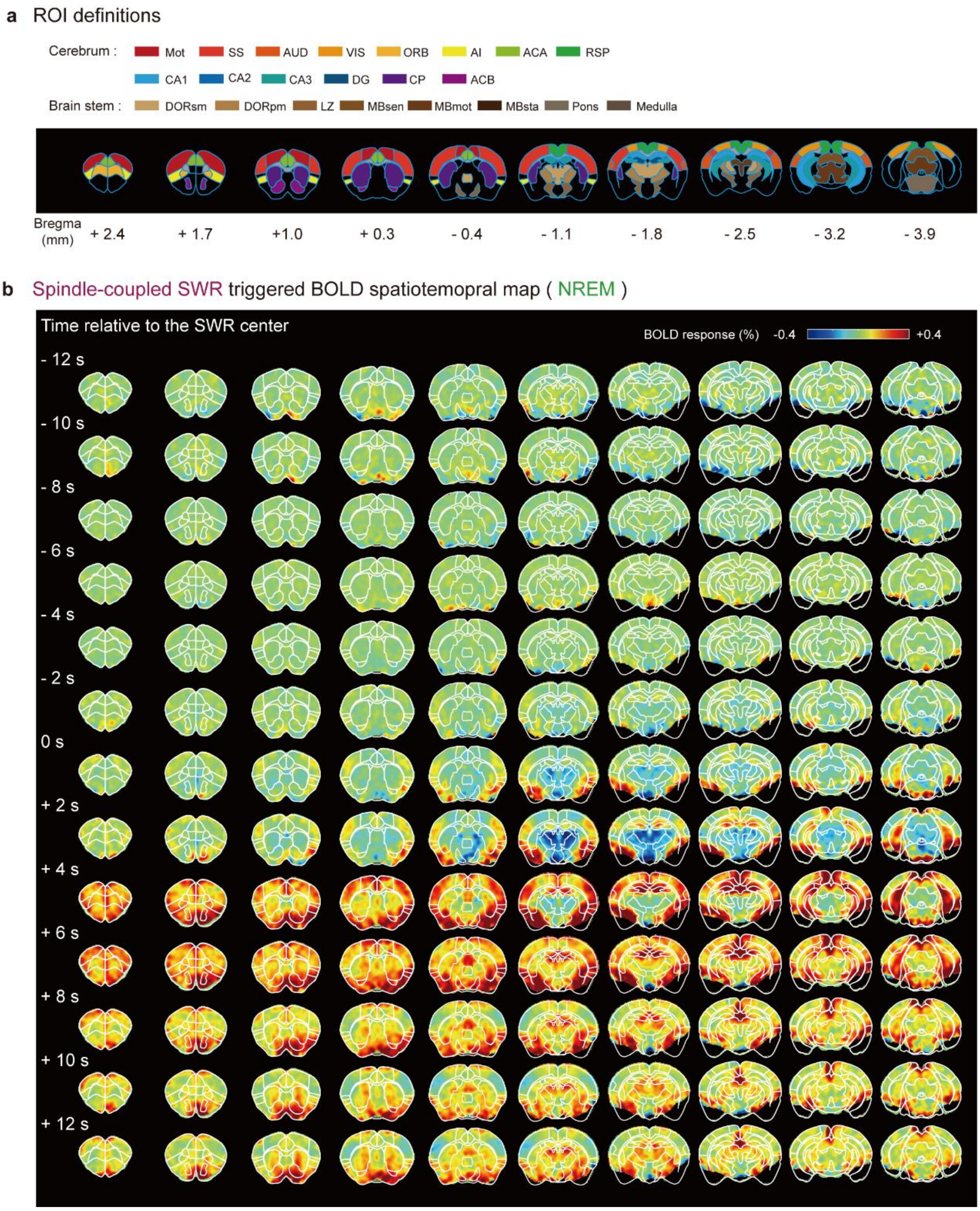

Supplementary Figure 22 | Related to Main Figure 7

Spindle-coupled SWR evoked BOLD signal dynamics in NREM state.

(a) ROI definitions derived from CCFv3 Allen mouse brain atlas. Abbreviations were list in Supplementary Data 1.

(b) Spatiotemporal BOLD signals evoked by the spindle-coupled SWR event in NREM state (6271 epochs). The number of event epochs were counted under the sampling rate of 0.5 Hz (fMRI repetition time). More quantitative ROI-wise evaluation was shown in Supplementary Data 4.

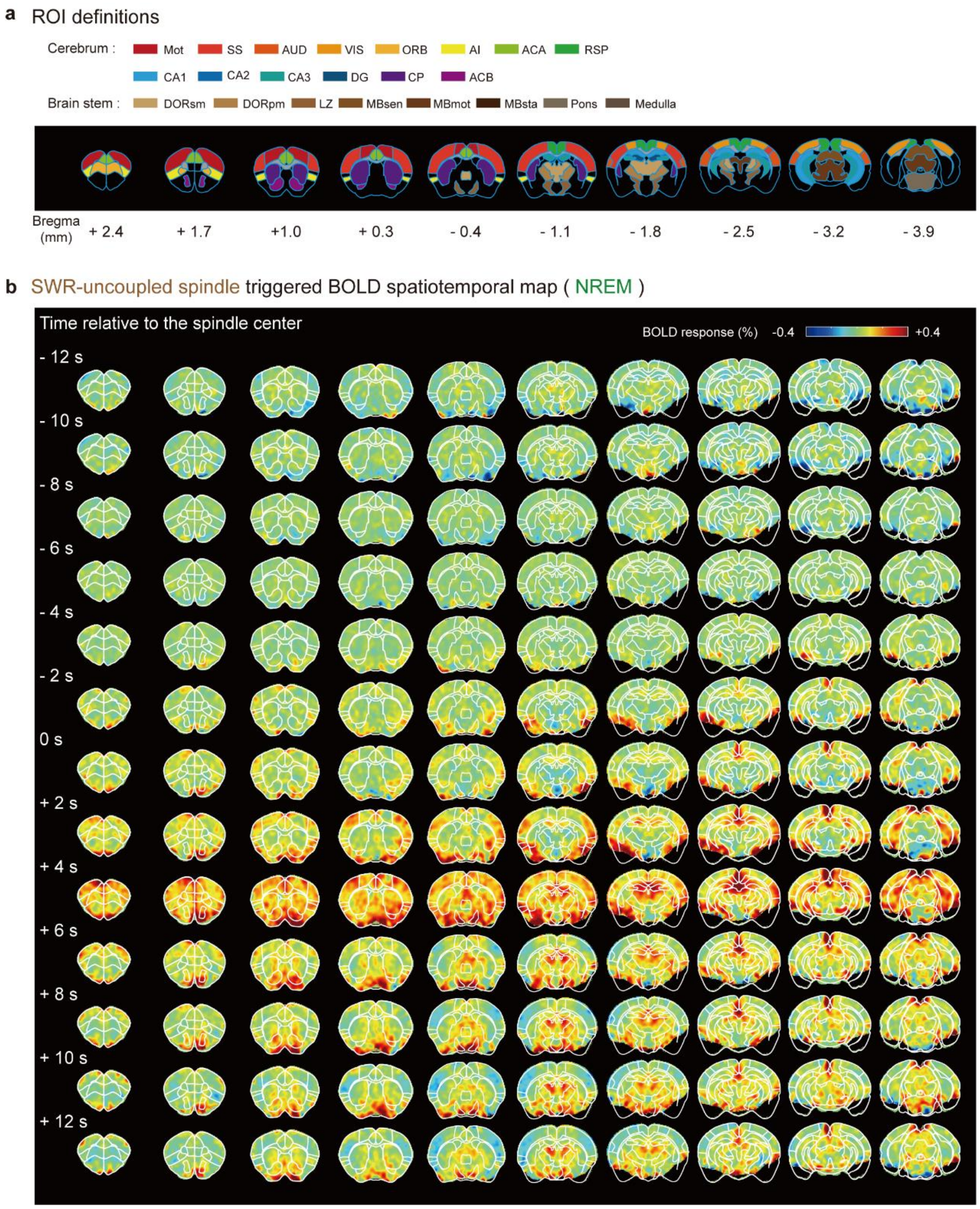

Supplementary Figure 23 | Related to Main Figure 7

SWR-uncoupled spindle evoked BOLD signal dynamics in NREM state.

(a) ROI definitions derived from CCFv3 Allen mouse brain atlas. Abbreviations were list in Supplementary Data 1.

(b) Spatiotemporal BOLD signals evoked by the SWR-uncoupled spindle event in NREM state (n=4288 epochs). The number of event epochs were counted under the sampling rate of 0.5 Hz (fMRI repetition time). More quantitative ROI-wise evaluation was shown in Supplementary Data 4.

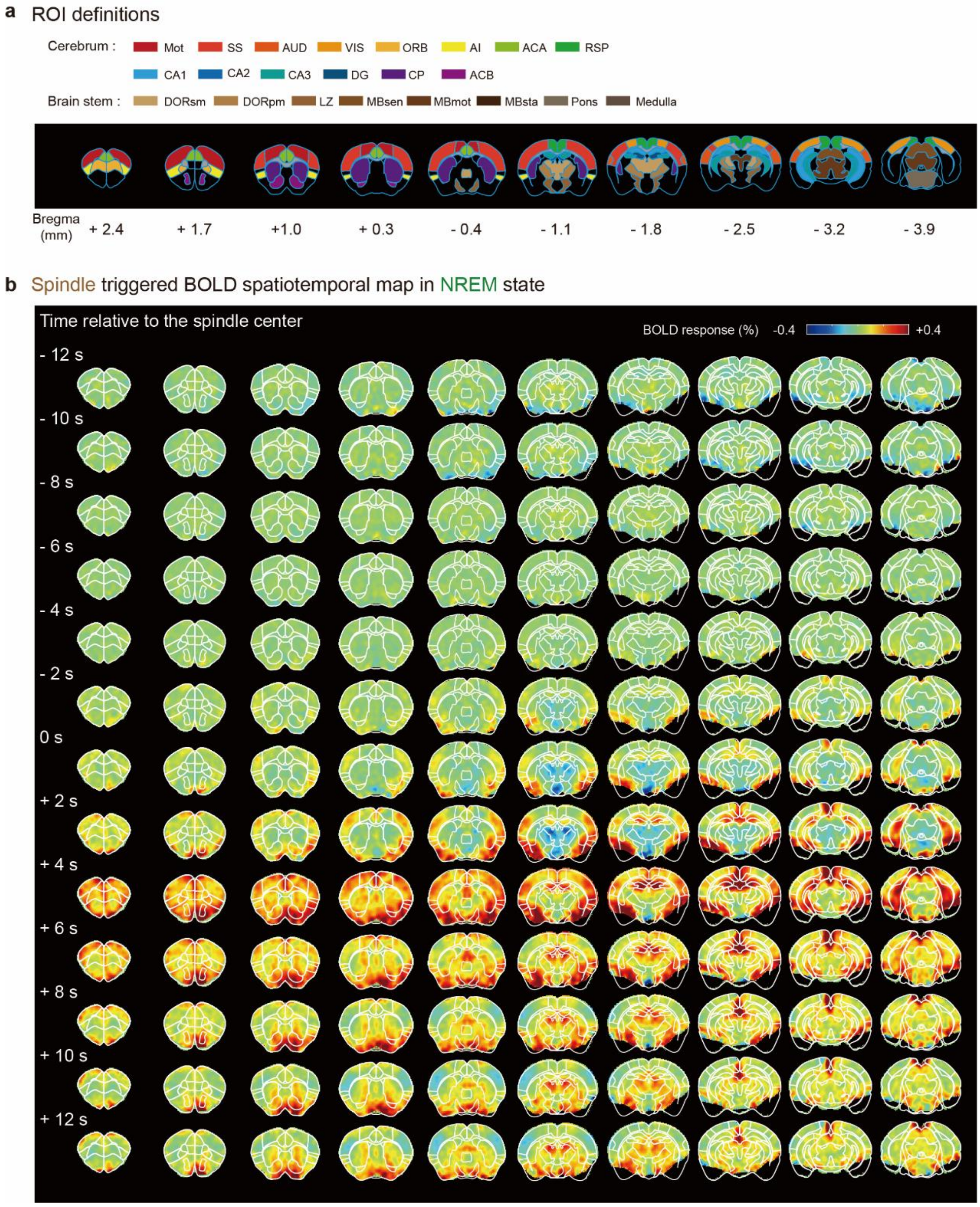

Supplementary Figure 24 | Related to Main Figure 7

Spindle evoked BOLD signal dynamics in NREM state.

(a) ROI definitions derived from CCFv3 Allen mouse brain atlas. Abbreviations were list in Supplementary Data 1.

(b) Spatiotemporal BOLD signals evoked by the spindle event in NREM state (n=10559 epochs). The number of event epochs were counted under the sampling rate of 0.5 Hz (fMRI repetition time). More quantitative ROI-wise evaluation was shown in Supplementary Data 4.

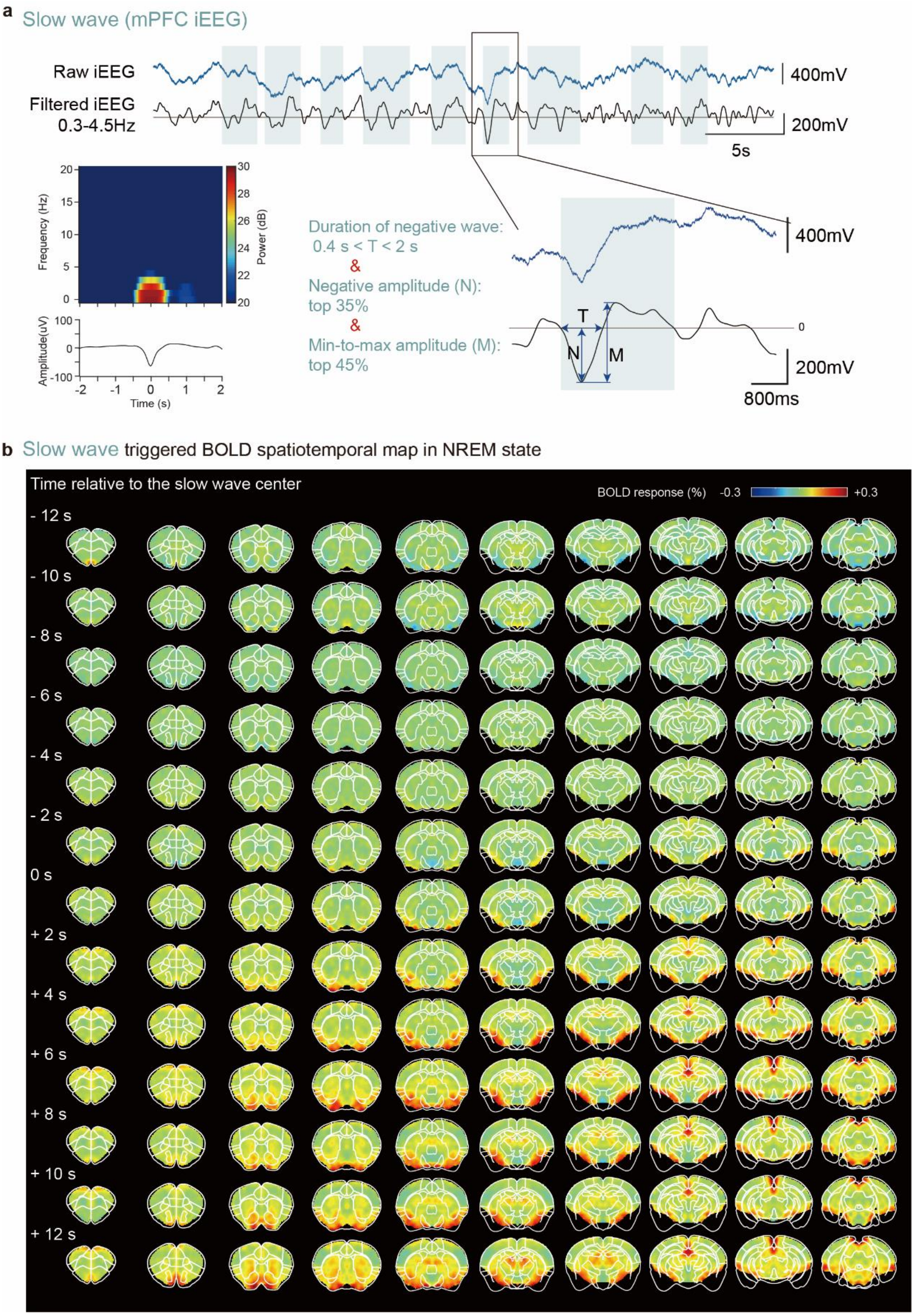

Supplementary Figure 25

Slow wave identifications and slow wave evoked BOLD signal dynamics.

(a) The procedure for extracting slow wave events. Raw iEEG signals (blue curves) in mPFC were bandpass filtered (black curves, 0.3-4.5Hz). A slow wave was detected in NREM state if the following three criteria were all fulfilled: (1) the interval (T) of negative wave between 0.4 and 2.0

s; (2) top 35% negative amplitude (N) and (3) top 45% negative-to-positive peak-to-peak amplitude (M). Slow wave onset and offset was defined by the time of the first and third zero crossing, respectively. Light blue shade represented the identified slow wave events. Lower left panel, averaged power spectrogram and time series of slow wave events.

(b) Spatiotemporal BOLD signals evoked by the slow waves (12599 epochs) in NREM state. The number of event epochs were counted under the sampling rate of 0.5 Hz (fMRI repetition time).
