# Supplementary material for: Sleep fMRI with simultaneous electrophysiology at 9.4 T in male mice
Source: Nat Commun. 2023 Mar 24;14:1651. doi: 10.1038/s41467-023-37352-9 (PMC10039056; doi:10.1038/s41467-023-37352-9)
Supplement: Supplementary file 3 — Description of Additional Supplementary Files [file 41467_2023_37352_MOESM3_ESM.pdf]

Description of Additional Supplementary Files

File Name: Supplementary Data 1
Description: The abbreviations list of brain regions name.

File Name: Supplementary Data 2
Description: Related to Main Figure 4. The first 100 principal components (PCs) of BOLD signals.

File Name: Supplementary Data 3
Description: Related to Supplementary Figure 8-11. The quantitative results of spatiotemporal BOLD signal variations during “AW to NREM”, “NREM to AW”, “NREM to REM”, “REM to AW” transition processes.

File Name: Supplementary Data 4
Description: Related to Supplementary Figure 17-18, 21-24. The quantitative ROI-wise evaluation of spatiotemporal BOLD signals evoked by SWRs or spindles. This file contains SWRs evoked BOLD signals in AW and NREM states, spindles evoked BOLD signal, SWRs-uncoupled spindles evoked BOLD signals, spindle uncoupled SWRs evoked BOLD signal and spindle coupled SWRs evoked BOLD signal.

File Name: Supplementary Data 5
Description: Related to Supplementary Figure 1. This file listed interpolated channels for each session.

File Name: Supplementary Data 6
Description: This file listed selected channels for the purpose of brain state classification in each session.
