# Supplementary material for: Sleep fMRI with simultaneous electrophysiology at 9.4 T in male mice
Source: Nat Commun. 2023 Mar 24;14:1651. doi: 10.1038/s41467-023-37352-9 (PMC10039056; doi:10.1038/s41467-023-37352-9)
Supplement: Supplementary file 8 — Supplementary Data 5 [file 41467_2023_37352_MOESM8_ESM.docx]

Interpolated channels:

| Session1 | Mouse1_day1 | Ch2, ch12 |
| --- | --- | --- |
| Session2 | Mouse1_day2 | Ch2, ch3 |
| Session3 | Mouse1_day3 | Ch2, ch12 |
| Session4 | Mouse2_day1 | Ch12 |
| Session5 | Mouse2_day2 | Ch12 |
| Session6 | Mouse3_day1 | Ch12 |
| Session7 | Mouse4_day1 | Ch2, ch12 |
| Session8 | Mouse4_day2 | Ch12 |
| Session9 | Mouse5_day1 | Ch5, ch10-12 |
| Session10 | Mouse6_day1 | \ |
| Session11 | Mouse6_day2 | Ch12 |
| Session12 | Mouse7_day1 | Ch12 |
| Session13 | Mouse8_day1 | Ch2, ch12 |
| Session14 | Mouse9_day1 | Ch12 |
| Session15 | Mouse9_day2 | Ch12 |
| Session16 | Mouse9_day3 | \ |
| Session17 | Mouse10_day1 | Ch12 |
| Session18 | Mouse10_day2 | Ch12 |
| Session19 | Mouse10_day3 | Ch12 |
| Session20 | Mouse11_day1 | Ch2 |
| Session21 | Mouse11_day2 | Ch2, ch3 |
| Session22 | Mouse12_day1 | \ |
| Session23 | Mouse13_day1 | \ |
| Session24 | Mouse13_day2 | \ |
| Session25 | Mouse13_day3 | Ch2, ch3 |
| Session26 | Mouse14_day1 | \ |
| Session27 | Mouse14_day2 | \ |
