# Supplementary material for: Sleep fMRI with simultaneous electrophysiology at 9.4 T in male mice
Source: Nat Commun. 2023 Mar 24;14:1651. doi: 10.1038/s41467-023-37352-9 (PMC10039056; doi:10.1038/s41467-023-37352-9)
Supplement: Supplementary file 9 — Supplementary Data 6 [file 41467_2023_37352_MOESM9_ESM.docx]

| Session1 | Ch10 |
| --- | --- |
| Session2 | Ch10 |
| Session3 | Ch10 |
| Session4 | Ch10 |
| Session5 | Ch10 |
| Session6 | Ch10 |
| Session7 | Ch13 |
| Session8 | Ch16 |
| Session9 | Ch2 |
| Session10 | Ch6 |
| Session11 | Ch2 |
| Session12 | Ch10 |
| Session13 | Ch8 |
| Session14 | Ch10 |
| Session15 | Ch10 |
| Session16 | Ch10 |
| Session17 | Ch8 |
| Session18 | Ch5 |
| Session19 | Ch5 |
| Session20 | Ch10 |
| Session21 | Ch16 |
| Session22 | Ch8 |
| Session23 | Ch13 |
| Session24 | Ch13 |
| Session25 | Ch13 |
| Session26 | Ch2 |
| Session27 | Ch2 |
