# Supplementary material for: Sleep fMRI with simultaneous electrophysiology at 9.4 T in male mice
Source: Nat Commun. 2023 Mar 24;14:1651. doi: 10.1038/s41467-023-37352-9 (PMC10039056; doi:10.1038/s41467-023-37352-9)
Supplement: Supplementary file 10 — Reporting Summary [file 41467_2023_37352_MOESM10_ESM.pdf]

nature portfolio

Corresponding author(s): Zhifeng Liang

Last updated by author(s): Mar 6, 2023

# Reporting Summary

Nature Portfolio wishes to improve the reproducibility of the work that we publish. This form provides structure for consistency and transparency in reporting. For further information on Nature Portfolio policies, see our Editorial Policies and the Editorial Policy Checklist.

## Statistics

For all statistical analyses, confirm that the following items are present in the figure legend, table legend, main text, or Methods section.

| n/a | Confirmed | |
|---|---|---|
| ☐ | ☒ | The exact sample size (*n*) for each experimental group/condition, given as a discrete number and unit of measurement |
| ☐ | ☒ | A statement on whether measurements were taken from distinct samples or whether the same sample was measured repeatedly |
| ☐ | ☒ | The statistical test(s) used AND whether they are one- or two-sided<br>*Only common tests should be described solely by name; describe more complex techniques in the Methods section.* |
| ☒ | ☐ | A description of all covariates tested |
| ☐ | ☒ | A description of any assumptions or corrections, such as tests of normality and adjustment for multiple comparisons |
| ☐ | ☒ | A full description of the statistical parameters including central tendency (e.g. means) or other basic estimates (e.g. regression coefficient) AND variation (e.g. standard deviation) or associated estimates of uncertainty (e.g. confidence intervals) |
| ☐ | ☒ | For null hypothesis testing, the test statistic (e.g. *F*, *t*, *r*) with confidence intervals, effect sizes, degrees of freedom and *P* value noted<br>*Give P values as exact values whenever suitable.* |
| ☒ | ☐ | For Bayesian analysis, information on the choice of priors and Markov chain Monte Carlo settings |
| ☒ | ☐ | For hierarchical and complex designs, identification of the appropriate level for tests and full reporting of outcomes |
| ☐ | ☒ | Estimates of effect sizes (e.g. Cohen's *d*, Pearson's *r*), indicating how they were calculated |

*Our web collection on statistics for biologists contains articles on many of the points above.*

## Software and code

Policy information about availability of computer code

| | |
|---|---|
| Data collection | ParaVision 6.0.1 for MRI acquisitions; Synapse software version 96(from Tucker-Davis Technologies) for electrophysiological signal recordings |
| Data analysis | MATLAB 2020a (MathWorks, Natick, MA), SPM12 (http://www.fil.ion.ucl.ac.uk/spm/) and FMRIB(FASTR) in EEGLAB (https://fsl.fmrib.ox.ac.uk/eeglab/fmribplugin/) for fMRI and ECoG/LFP processing; Chronux toolbox (http://chronux.org/) for power spectrum analysis; matlab LSTM toolbox for prediction analysis. |

For manuscripts utilizing custom algorithms or software that are central to the research but not yet described in published literature, software must be made available to editors and reviewers. We strongly encourage code deposition in a community repository (e.g. GitHub). See the Nature Portfolio guidelines for submitting code & software for further information.

## Data

Policy information about availability of data

All manuscripts must include a data availability statement. This statement should provide the following information, where applicable:

- Accession codes, unique identifiers, or web links for publicly available datasets
- A description of any restrictions on data availability
- For clinical datasets or third party data, please ensure that the statement adheres to our policy

The manuscript released a simultaneous electrophysiological-fMRI dataset, which is open public available at https://www.doi.org/10.12412/

BSDC.1668502646.20001. Codes used in this study are available at https://github.com/TrangeTung/Mouse_Sleep_fMRI.

## Human research participants

Policy information about studies involving human research participants and Sex and Gender in Research.

| | |
|---|---|
| Reporting on sex and gender | n/a, This study did not involve human participants. |
| Population characteristics | n/a |
| Recruitment | n/a |
| Ethics oversight | n/a |

Note that full information on the approval of the study protocol must also be provided in the manuscript.

# Field-specific reporting

Please select the one below that is the best fit for your research. If you are not sure, read the appropriate sections before making your selection.

☒ Life sciences ☐ Behavioural & social sciences ☐ Ecological, evolutionary & environmental sciences

For a reference copy of the document with all sections, see nature.com/documents/nr-reporting-summary-flat.pdf

# Life sciences study design

All studies must disclose on these points even when the disclosure is negative.

| | |
|---|---|
| Sample size | No sample-size calculation was performed.<br>This sample size in our study (27 mice) have shown to be sufficient to investigate the properties of sleep stages (e.g. 7 rats with 35 sleep sessions, Bergel A,, Tanter M, Cohen I, et. al., Nat Commun, 2018; 20 mice, Patrick J Drew, et. al., eLife, 2020; 6 mice, Wang Z, Zhang S, Xu M, et. al., Nat Commun. 2022). |
| Data exclusions | No data was excluded for analysis. |
| Replication | n/a<br>For simultaneous ECoG/LFP-fMRI data collection, identical imaging and recording procedures were applied to mice, which formed the dataset in this manuscript. For the replication of scientific insights, we replicated same analysis using the ECOG-fMRI and LFP-fMRI subset (Fig.2-3, Supplementary Fig 2), showing high similarity between ECOG-fMRI and LFP-fMRI subset. The exact number of mice and recording sessions in the experiment was reported in the text and/or figure legends. |
| Randomization | n/a<br>There are no experimental and control groups in our study. |
| Blinding | n/a<br>There are no experimental and control groups in our study. Blinding was not necessary, since all mouse individuals were passed through identical processing pipelines. |

# Reporting for specific materials, systems and methods

We require information from authors about some types of materials, experimental systems and methods used in many studies. Here, indicate whether each material, system or method listed is relevant to your study. If you are not sure if a list item applies to your research, read the appropriate section before selecting a response.

### Materials & experimental systems

| n/a | Involved in the study | |
|---|---|---|
| ☒ | ☐ | Antibodies |
| ☒ | ☐ | Eukaryotic cell lines |
| ☒ | ☐ | Palaeontology and archaeology |
| ☐ | ☒ | Animals and other organisms |
| ☒ | ☐ | Clinical data |
| ☒ | ☐ | Dual use research of concern |

### Methods

| n/a | Involved in the study | |
|---|---|---|
| ☒ | ☐ | ChIP-seq |
| ☒ | ☐ | Flow cytometry |
| ☐ | ☒ | MRI-based neuroimaging |

## Animals and other research organisms

Policy information about studies involving animals; ARRIVE guidelines recommended for reporting animal research, and Sex and Gender in Research

| | |
|---|---|
| Laboratory animals | C57BL/6J mice (8-10 weeks of age) |
| Wild animals | This study did not involve wild animals. |
| Reporting on sex | Male |
| Field-collected samples | The study did not involve samples collected from the field. |
| Ethics oversight | All animal experiments were approved by the Animal Care and Use Committee of the Institute of Neuroscience, Chinese Academy of Sciences, Shanghai, China. |

Note that full information on the approval of the study protocol must also be provided in the manuscript.

## Magnetic resonance imaging

### Experimental design

| | |
|---|---|
| Design type | resting-state |
| Design specifications | each session contains one scan, each scan lasts four hours |
| Behavioral performance measures | respiratory signal was also recorded. |

### Acquisition

| | |
|---|---|
| Imaging type(s) | Functional (resting state fMRI); Structural (T2-weighed anatomical image) |
| Field strength | 9.4T, Bruker |
| Sequence & imaging parameters | A T2 weighted RARE anatomical image (TR: 3200 ms; TE: 34 ms; matrix size: 256×128; FOV: 18×9 mm2; slice thickness: 400 µm; resolution: 70×70 µm2) was acquired for coregistration purpose. After local shimming using Mapshim, functional images were acquired using single-shot echo planar imaging (EPI) with the following parameters: TR 2000 ms, TE 14 ms, FA 70°, matrix size 90×45 µm2, nominal in-plane resolution 200×200 µm2, slice thickness 400 µm, slices number 22, 7200 EPI volumes (4 hours). |
| Area of acquisition | Whole brain |

Diffusion MRI ☐ Used ☒ Not used

### Preprocessing

| | |
|---|---|
| Preprocessing software | MATLAB 2020a and SPM12 for fMRI processing |
| Normalization | The fMRI data was coregistered to the T2 anatomical images (spm12, coregistration), which were further coregistered to a template (spm12, oldnormalize). |
| Normalization template | Allen template(https://atlas.brain-map.org/) |
| Noise and artifact removal | The resting state fMRI data were further regressed by "6 rp + 6 Δrp + 40 PCs" nuisance signals to reduce motion artifacts, including 6 head motion parameters, their 1st order derivatives and 40 non-brain tissue based principal components (PCs). |
| Volume censoring | No fMRI volume was censored during fMRI preprocessing. |

### Statistical modeling & inference

| | |
|---|---|
| Model type and settings | GLM based statistical analysis was conducted using the mouse-specific HRF. |
| Effect(s) tested | Standard first level analysis was done for individual EPI scans. For second level analysis, flexible factorial model (brain states and mouse individuals) was conducted to generate the activation maps. |

Specify type of analysis: ☒ Whole brain ☐ ROI-based ☐ Both

| | |
|---|---|
| Statistic type for inference (See Eklund et al. 2016) | t-test |

Correction | FDR corrected $p < 0.05$

## Models & analysis

| n/a | Involved in the study |
|---|---|
| ☒ | ☐ Functional and/or effective connectivity |
| ☒ | ☐ Graph analysis |
| ☐ | ☒ Multivariate modeling or predictive analysis |

Multivariate modeling and predictive analysis

GLM based statistical analysis was conducted using the mouse-specific HRF, in which NREM and REM states were set as the predictors and thus the AW state was used implicitly as the baseline. Standard first level analysis was done for individual EPI scans. For second level analysis, flexible factorial model (brain states and mouse individuals) was conducted to generate the activation maps.
The long short-term memory (LSTM) recurrent neural networks (RNNs) model was built to predict the brain state based on its functional profile, i.e., tPCs, and their temporal dependency of BOLD signals on its preceding time points. The LSTM RNNs used in this study contains one (or two or three) hidden LSTM layer(s) and one fully connected layer. Multiple hidden LSTM layers could be used to encode the functional information with temporal dependency for each time point, and the fully connected layer was used to learn a mapping between the learned feature representation and brain states. The time series of each principal component (tPCs) were used as the input of the LSTM RNNs model to predict their corresponding brain states.
